# TLR9 and RIG-I Signaling in Human Endocervical Epithelial Cells Modulates Inflammatory Responses of Macrophages and Dendritic Cells *In Vitro*

**DOI:** 10.1371/journal.pone.0083882

**Published:** 2014-01-07

**Authors:** Ameya Sathe, Kudumula Venkata Rami Reddy

**Affiliations:** Division of Molecular Immunology and Microbiology (MIM), National Institute for Research in Reproductive Health (NIRRH), Indian Council of Medical Research, Mumbai, India; University of Missouri-Kansas City, United States of America

## Abstract

The innate immune system has evolved to recognize invading pathogens through pattern recognition receptors (PRRs).Among PRRs, Toll like receptors (TLRs 3, 7/8,9) and RIG-I like receptors (RLRs) have been shown to recognize viral components. Mucosal immune responses to viral infections require coordinated actions from epithelial as well as immune cells. In this respect, endocervical epithelial cells (EEC's) play an important role in initiating innate immune responses via PRRs. It is unknown whether EEC's can alter immune responses of macrophages and dendritic cells (DC's) like its counterparts in intestinal and respiratory systems. In this study, we show that endocervical epithelial cells (End1/E6E7) express two key receptors, TLR9 and RIG-I involved in anti-viral immunity. Stimulation of End1/E6E7 cells lead to the activation of NF-κB and increased secretion of pro-inflammatory cytokines, IL-6 and IL-8. Polarized End1/E6E7 cells responded to apical stimulation with ligands of TLR9 and RIG-I, CpG-ODN and Poly(I:C)LL respectively, without compromising End1/E6E7 cell integrity. At steady state, spent medium from End1/E6E7 cells significantly reduced secretion of pro-inflammatory cytokines from LPS treated human primary monocyte derived macrophages (MDMs) and DC:T cell co-cultures. Spent medium from End1/E6E7 cells stimulated with ligands of TLR9/RIG-I restored secretion of pro-inflammatory cytokines as well as enhanced phagocytosis and chemotaxis of monocytic U937 cells. Spent medium from CpG-ODN and Poly(I:C)LL stimulated End1/E6E7 cells showed significant increased secretion of IL-12p70 from DC:T cell co-cultures. The anti-inflammatory effect of spent media of End1/E6E7 cell was observed to be TGF-β dependent. In summary, the results of our study indicate that EEC's play an indispensable role in modulating anti-viral immune responses at the female lower genital tract.

## Introduction

Cervico-vaginal epithelium of the lower female reproductive tract (FRT) is constantly bombarded with a variety of both innocuous (eg: commensal flora) and pathogenic microorganisms (eg: virus, bacteria and parasites) [1]. Cervico-vaginal epithelial cells (CVECs), which line the luminal surface of the vaginal epithelium, are the first cell type to be activated after an initial insult by invading pathogens. CVECs respond to “danger signals” and produce an array of innate immune factors such as complement, pro-inflammatory mediators, adhesion molecules and anti-viral factors, which allow CVECs to communicate with the immune system [2], [3]. Several studies have shown that CVECs, macrophages, and dendritic cells (DC's) execute immunological functions by expressing pattern recognition receptors (PRRs) such as Toll-like receptors (TLRs), RNA helicases like receptors (RLR) [4] and NOD-like receptors (NLR) [5]. However, the mechanism by which these cells function together is limited.

TLR's (eg: TLR3, 9, 7/8) and cytoplasmic RNA helicase, retinoic acid-inducible gene-I (RIG-I) have been shown as important sensors of innate immunity to viruses and their components including nucleic acids and envelope glycoproteins. These three R's (TLRs, RLR's and NLR's) known to be expressed in different intracellular compartments [6], [7]. Recognition of viral nucleic acids by these receptors induces type-I interferon, inflammatory cytokines/chemokines which are necessary to eliminate viral pathogens [2], [3], [4], [8], [9]. TLR9, for example, patrols the endosomal compartments of cells and recognizes unmethylated deoxycytidyl-phosphate-deoxyguanosine (CpG) dinucleotides that are commonly found in bacterial and viral genomes (eg:HSV-2) [6], [10]. Others have shown that TLR7/8 recognizes imiquidizalones such as imiquimod, resiquimodand single stranded RNA (ssRNA) [11], whereas TLR3 recognizes viral double-stranded RNA (dsRNA) which found during viral replication [12] and RIG-I survey for the presence of viral dsRNAs within the cytoplasm [6].

In addition to the direct activation of dendritic cells (DC's) byTLR9 and TLR3, recent studies showed that DC's require TLR-dependent instructive signals from the infected cells in order to generate anti-viral immune responses *in vivo* [13], [14], [15]. From these studies, it is understood that clearance of pathogens requires coordinated immune responses involving epithelial as well as immune cells. Recognizing that human endocervical epithelial cells (EEC's) act as a physical barrier to encounter potential pathogens, the present study was undertaken to define the extent to which the key intracellular PRRs, TLR9 and RIG-I are responsive to their ligands *in vitro* and the mechanism by which these cells work together with immune cells under steady state and inflammatory conditions. The objectives of the present study are: 1) to determine if human endocervical epithelial cells (End1/E6E7) express TLR9 and RIG-I receptors, 2) to determine whether End1/E6E7 cells respond to ligands of TLR9 and RIG-I and 3) to decipher the effect of spent media obtained from unstimulated and TLR9 and RIG-I ligand stimulated End1/E6E7 cells on inflammatory responses in human primary monocyte derived macrophages (MDMs) and monocyte derived dendritic cell's (MDDCs).

The results of the present study demonstrated that End1/E6E7 cells constitutively express TLR9 and RIG-I intracellularly and the ligands of these receptors, CpG-ODN (CpG – oligodeoxynucleotide) and Poly(I:C)LL respectively, induced the activation of pro-inflammatory cytokines, IL-6, IL-8 and GM-CSF production via NF-κB signaling. Under steady state condition, End1/E6E7 cells inhibited secretion of pro-inflammatory cytokines from MDMs and MDDCs. This effect was mediated by End1/E6E7 cells derived TGF-β, since neutralization of TGF-β restored TNF-α secretion by macrophages. On the contrary, stimulation of End1/E6E7 cells with CpG-ODN and Poly(I:C)LL reduced TGF-β levels, consequently secretions of End1/E6E7 cells enhanced inflammation. To the best of our knowledge, for the first time we demonstrated TLR9 and RIG-I are functional in End1/E6E7 cells and play critical role in antiviral immune responses. The results of this study would aid in understanding the cross-talk between epithelial pattern recognition and inflammatory responses of macrophages and DC's.

## Materials and Methods

### Ethics Statement

The study design and procedure was approved by the NIRRH Ethics Committee (D/IECCR/181/2010) for Clinical Studies, NIRRH, Mumbai. Written informed consent was obtained from healthy individuals prior to their participation. After obtaining informed consent, 10 ml of blood samples were collected from healthy adult volunteers (age 21–40 years).

### Reagents

TLR9 and RIG-I agonists {(Human CpG-oligodeoxynucleotide (CpG-ODN2006)} (henceforth referred as CpG-ODN)and Poly(I:C)LMW (low molecular weight)-Lyovec {henceforth referred as Poly(I:C)LL} respectively, were purchased from Invivogen and used at a concentration of 10 µg/ml. LPS was derived from *Escherichia coli* O55:B5 procured from Sigma-Aldrich. Following antibodies were procured from vendors mentioned in parentheses: TGF-β neutralizing monoclonal antibody (Peprotech); rabbit polyclonal against phospho p65, mouse monoclonal against β-actin, rabbit polyclonal against RIG-I (Abcam); mouse monoclonal against TLR9 (Imgenex), FITC and HRP labeled secondary antibodies (Sigma-Aldrich). Unless otherwise specified, all other reagents are of high quality grade were procured from local suppliers.

### Cell lines and culture conditions

#### End1/E6E7 cell line

Human End1/E6E7 cell line is a well-differentiated endocervical epithelial cell line (referred as End1/E6E7 cells) derived from normal endocervical epithelium. The cell line was developed by immortalizing with human papillomavirus-16/E6E7 by Dr. Raina Fichorova, Brigham Women's Hospital, Harvard Medical School, Boston, MA, USA and has been described previously [16]. This cell line was obtained as a gift from Dr. Fichorova.

Mycoplasma free End1/E6E7 cells were maintained in keratinocyte serum-free medium (KSFM, Life Technologies) supplemented with the provided bovine pituitary extract (BPE, 50 µg/ml) and recombinant epidermal growth factor (EGF, 0.1 ng/ml), and 0.4 mM CaCl_2_ (KSFM growth medium) and Pen/Strep. Cells were maintained at 37°C in a humidified atmosphere containing 5% CO_2_. These cells were periodically checked and found to be free of mycoplasma.

#### U937 cell line

U937 cell line was procured from National Center for Cell Science (NCCS), Pune, India and cultured as per the instructions of the suppliers.

### Analysis of LPS contamination in TLR9 and RIG-I ligands preparation

To check whether inflammatory stimulus elicited by End1/E6E7 cells in response to CpG-ODN and Poly (I:C)LL treatment were not the result of contamination with LPS, CpG-ODN and Poly (I:C)LL samples were analyzed by Limulus amebocyte lysate (LAL) assay as per the manufacturers protocol (Charles River, USA). We also further ensured the responses observed were specific to CpG-ODN and Poly (I:C)LL by stimulating End1/E6E7 cells at 37°C for 24 hrs with CpG-ODN (10 µg/mL)+LPS (0.05 ng/mL) and Poly (I:C)LL (10 µg/mL)+LPS (0.05 ng/mL) and by collecting the spent media for IL-8 levels by ELISA as described elsewhere.

### Cell viability

In all experiments, microscopic examination of trypan blue exclusion and mitochondrial respiration (an indicator of cell viability) was determined by the mitochondrion-dependent reduction of MTT {3-(4,5-dimethylthiazol-2-yl)-2,5-diphenyltetrazolium bromide} (Sigma-Aldrich) to formazan, which was carried out according to the manufacturer's instructions. Briefly, End1/E6E7 cells were incubated with media containing CpG-ODN or Poly(I:C)LL (10 µg/mL) in 96-well plates for 24 hrs. The cells were then incubated with MTT (0.5 mg/ml) for 3 hrs at 37°C. Upon formation of formazan crystals, culture medium was removed by aspiration and cells were solubilized in dimethyl sulfoxide (DMSO) (100 µl). The extent of MTT reduction to formazan within cells was quantitated by measuring optical density at 550 nm using a microplate reader (BioTek Instruments, Germany).

### qPCR analysis

To determine expression of TLR9 and RIG-I at basal and stimulated conditions, End1/E6E7 cells were grown in KSFM in 24 well plates. Cells were stimulated with fresh media containing CpG-ODN or Poly(I:C)LL (10 µg/mL) for 6 hrs at 37°C before analyzing TLR9 and RIG-I expression by a battery of assays that include qPCR, RT-PCR, Western blot, flow cytometry and indirect immunofluorescence. The concentration of PRR ligands used in this study was based on *in vitro* studies published previously [3], [17].

To determine TLR9, RIG-I, IL-6 and IL-8 mRNA expression, End1/E6E7 cells were stimulated with CpG-ODN (10 µg/ml), Poly (I: C)LL (10 µg/ml) for 6 hrs. Total RNA was extracted using Trizol reagent according to the manufacturer's recommendations (Invitrogen). One µg of total RNA was reverse-transcribed using the iScript first strand cDNA synthesis kit (Bio-Rad, USA). Relative mRNA expression levels of genes of interest were measured using SYBR Green chemistry on the Bio-Rad CFX 96 well real time PCR machine (Bio-Rad). Sequences of primers used for analysis are given in Table 1. Unstimulated cells served as controls. Data was analyzed with untreated values set as calibrator and expression normalized to 18S rRNA. Fold change was calculated by 2^−ΔΔCt^ method.

**Table 1:** List of primer sequences used for qPCR in the study.

Gene	Sequence 5′→3′	Accession No.	Product Size (bp)	Annealing Temp (°C)
IL-6	**F**: GAAAGCAGCAAAGAGGC **R**: GAAGCATCCATCTTTTTCA	NM_000600.3	72	62
IL-8	**F**: CACCGGAAGGAACCATCTCACT **R**: TGCACCTTCACASCAGAGCTGCA	NM_000584.3	101	62
TLR9	**F**: GCGACCAGGCTCCCGAAGG **R**: GTGTCCTTTGCCCACCTGTCTC	NM_017442.3	50	63
RIG-I	**F**: TGTGGGCAATGTCATCAAA **R**: GAAGCACTTGCTACCTCTTGC	NM_014314.3	63	64
18S rRNA	**F**: GTAACsCCGTTGAACCCCATT**R**: CCATCSCAATCGGTAGTAGCG	NR_003286.2	151	60

### RT-PCR analysis

Total cellular RNA was extracted from control and ligand induced End1/E6E7 cells by TRIZOL reagent (Invitrogen) according to the manufacturer's protocol and treated with DNase (Ambion). After testing the RNA for integrity and possible contaminants, the RNA and no-RT controls were reverse transcribed using the iScript First-Strand Synthesis System for RT-PCR (Bio-Rad) following the manufacturer's protocol. PCR products of the expected size were generated with each primer pair. Specific primer sequences used were: TLR9 (397 bp) sense 5′-ATGAACTCCTTCTCCACAAG-3, anti-sense 5′-ACATTTGCCGAAGAGCCCTCAG-3′; RIG-I (154 bp) sense 5′-CTTGGCAGCCTTCCTGATTT-3′, anti-sense 5′- CTCAGCCCTCTTCAAAAACT-3′; and GAPDH (199 bp) sense 5′-CCATTCATTGAC CTCCACTACA-‘3, anti-sense 5′-CGTTGCTGACAATCTTGAGAGA-3′.

Thirty-five cycles of PCR amplification were performed on the PTC-100 Thermal Cycler (MJ Research, Inc.) as follows: denaturation, 94°C for 30 sec; annealing, 55°C for 30 sec; and extension, 72°C for 1 min. PCR products were separated on a 2% agarose gel with electrophoresis and visualized by ethidium bromide staining under UV illumination. The gels were scanned using a Gel Documentation System (Gel Doc 2000, Bio-Rad Laboratories) and intensity of the bands were quantified by ‘Quantity-One’ software.

### Flow cytometry analysis of TLR9 and RIG-I

For flow cytometric analysis of TLR9 and RIG-I expression, cells growing in culture were treated with TLR9 and RIG-I ligands as described above. After washing, cells were incubated for 1 hr at room temperature with anti-TLR9 or anti-RIG-I antibody (1∶1000) diluted in PBS with 1% FBS. After incubation, cells were washed thrice with PBS and then stained with FITC labeled goat anti-mouse (for TLR9) or goat anti-rabbit (for RIG-I). After incubation for 1 hr at RT, cells were washed thrice and resuspended in PBS in 1% FBS, and analyzed by flow cytometry (Becton Dickinson) available at the central equipment facility of NIRRH, Mumbai. For intracellular localization, same protocol was followed except cells were first treated with fixation buffer (4% paraformaldehyde) followed by permeabilization buffer (0.1% saponin), according to the manufacturer's protocol (Imgenex) before ligand treatment. The percentage positive cells and mean fluorescence were calculated. Data for 10,000 cells were collected for each sample. Secondary antibody alone was included as negative controls.

### Western blot analysis

To assess whether CpG-ODN and Poly(I:C)LL-induce TLR9 and RIG-I protein expression in End1/E6E7 cells, whole cell protein extracts were prepared from control and ligand treated cells with ice cold RIPA buffer supplemented with a protease inhibitor cocktail (Sigma-Aldrich). Cell lysates were centrifuged at 1000 g for 20 minutes at 4°C and supernatants were collected. Protein concentration was quantified with the BCA protein assay (Pierce, USA). Ten µg of protein from each sample was separated by 10% SDS-PAGE electrophoresis and proteins were transferred to polyvinylidenedifluoride (PVDF) membranes (Bio-Rad). The strips were blocked with 5% BSA in 0.1% TBST for 1 hr at RT and then probed with goat anti-mouse (for TLR9) or goat anti-rabbit (for TLR9) antibodies (1∶1000 dilution). The membranes were washed with 0.1% TBST buffer for 2 hrs followed by probing with horseradish peroxidase (HRP)-conjugated goat anti-mouse (for TLR9) or goat anti-rabbit (for RIG-I) secondary antibodies (1∶10000 dilution). The membranes were then washed 0.1% TBST for 3 hrs and bands were visualized using advance chemiluminescence detection system (ECL) (GE Healthcare, USA).

### Immunofluorescence analysis

End1/E6E7 cells were cultured on glass chamber slides (Fisher Scientific, USA) and were stimulated with CpG-ODN or Poly(I:C)LL (10 µg/ml each) for 6 hrs. The cells were washed three times with phosphate-buffered saline (PBS) and then fixed for 15 min in PBS with 4% paraformaldehyde. After washing with gentle shaking, the cells were permeabilized for 5 min with ice-cold methanol and washed. The fixed cells were then blocked in 2% BSA in PBS for 1 h at room temperature followed by incubation overnight at 4°C with mouse monoclonal anti-TLR9 or rabbit polyclonal anti-RIG-I antibody (1∶100) or rabbit polyclonal p65 NF-kB (Ser536) antibody (1∶100 dilution) (Abcam). After washing with PBS, slides were incubated for 1 h at RT in FITC-labeled secondary antibody (1∶500 dilutions). In between each step, the slides were washed 3 times for 5 min each in blocking solution. After counter staining with nuclear stains, propidium iodide (PI) (for TLR9 and RIG-I) or 4′,6-diamidino-2-phenylindole (DAPI) (for p65-NF-κB), slides were mounted in Vectashield mounting medium (Vector Laboratories, USA) and visualized under confocal laser scanning microscopy-LCSM (Zeiss, 510 Meta, Germany). Images were digitalized using CCD digital camera and Image-Pro Express Software at the central equipment facility of NIRRH, Mumbai.

### NF-κB ELISA

To determine whether CpG-ODN and Poly (I:C)LL has any effect on phosphorylated p65 NF-κB expression in End1/E6E7 cells, we used p65-NF-κB (Ser536) Sandwich ELISA kit (Cell Signaling). The treatment details are the same as described above for immunofluorescence analysis. After the treatment with CpG-ODN and Poly (I:C)LL (10 µg/ml for 30 min at 37°C), End1/E6E7 cells were lysed with hypotonic HEPES lysis buffer (pH 7.4) and centrifuged at 1000 g for 10 min at 4°C, supernatants were collected and used for the estimation of p65-NF-*κ*B as described earlier [18].

### Phagocytosis assay

Bacteria (*E.coli*) were labeled with fluorescein isothiocyanate (FITC). Briefly, *E.coli* cells were suspended in DPBS (pH 7.4) and centrifuged at RT for 5 min. The pellet was resuspended in 0.5 ml FITC solution (1 mg FITC/ml of buffer containing 50 mM sodium carbonate, 100 mM NaCl). The mixture was wrapped in aluminium foil to prevent bleaching by light and incubated at RT for 20 min. *E.coli* were washed three times with Dulbecco's Phosphate Buffered Saline (DPBS) containing 3 mM glucose, 0.5 mg/ml human serum albumin and 0.2 unit/ml aprotinin. The concentration of FITC labeled *E. coli* was adjusted to 2×10^8^ cfu/ml (OD of 0.1 at 600 nm). *E. coli* were stored at 4°C in dark for immediate use.

U937 cells were incubated at 37°C for 3 hrs with fresh media (control), untreated spent media (UT-SM) and spent media from End1/E6E7 cells treated with CpG-ODN (CpG-SM) or Poly(I:C)LL (Poly-SM). After washing, these cells were further incubated for 30 min. at 37°C with FITC labeled *E.coli* (ratio of U937 cells: *E.coli* was 1∶20) in a total volume of 1 ml in siliconized glass tubes. U937 cells not exposed to *E.coli* were handled identically to obtain background values. After incubation, 1 ml of ice-cold complete RPMI medium per ml was added, and centrifuged (110 g, 8 min) to separate phagocytic cells from free bacteria. Cells were washed twice in complete medium. Quantitative analysis of U937 cells that had internalized FITC labeled *E. coli* was performed on a FACS vantage flow cytometer (Becton Dickinson, USA) at the central equipment facility of NIRRH, Mumbai, India. The fluorescence signals for each *E.coli* cell present within U937 cells were detected through a 520 nm argon-ion laser. Plain U937 cells were included as appropriate negative control to rule out any nonspecific activity. The percentage of U937 cells with engulfed labeled *E.coli* was calculated using cell quest software (http://facs.scripps.edu).

### Chemotaxis (Transwell migration) assay

The effect of spent media obtained from CpG-ODN (CPG-SM) and Poly(I:C)-LL (Poly-SM) stimulated End1/E6E7 cells on chemotaxis of U937 cells was determined *in vitro* using Boyden chamber Transwell migration assay [18]. Briefly, the assay was carried out for 24 hrs at 37°C with 5% CO_2_. U937 cells migrated from upper chamber towards lower chamber were collected, fixed with 3.7% formaldehyde, and stained with a 0.2% crystal violet solution. Cells treated with 700 µl of 0.1 mM chemotactic peptide (N-formyl-methionyl-leucyl-phenylalanine (fMLP) in PBS-bovine serum albumin (BSA) were placed in the lower chamber and considered a positive control for cell migration. Non-migratory cells on the upper chamber of the membrane were removed with a cotton swab. For quantification, five randomly selected fields on the lower side of the membrane were counted through a 20× objective using computer-assisted microscope and analyzed with CellProfiler 2.0 cell image-analysis software.

### Determination of transepithelial resistance (TER)

Transepithelial electrical resistance (TER), the marker for intercellular tight junction integrity, provides an electrical measurement of barrier function and is negatively associated with permeability of a polarized epithelium [18]. To establish a polarized epithelial cell monolayer cultures with both apical and basolateral compartments, End1/E6E7 cells were seeded at 3×10^5^cells/per square centimeter were grown in Transwell inserts with a 6.5-mm diameter on collagen (type IV) coated polycarbonate culture inserts (Millipore, USA) having pore size of 0.4 µm, and incubated at 37°C in a humidified atmosphere of 5% CO_2_. The Transwell inserts were cultured for 8–10 days until End1/E6E7 cells established monolayers. Apical and basolateral compartments had 200 and 900 µl of complete medium respectively, and medium was changed every 2 days. Confluence of the cells was confirmed by measuring the TER (Millicell ERS Ohmmeter, Millipore). On day 8^th^ of culture, TER values stabilized and formed a confluent monolayer, exceeded the cut-off point of 150 Ω/cm^2^. As an indicator of tight junction formation of epithelial cell monolayers, TER was periodically assessed. The net TER was calculated by subtracting the background and multiplying the resistance (X) by the area (0.33 cm^2^) of the filter.

### Ligand treatment of polarized End1/E6E7 cells

In order to determine whether CpG-ODN and Poly (I:C)LL have any adverse effects on monolayer formation, End1/E6E7 cell confluent monolayers were treated for 24 hrs with these ligands (10 µg/mL each) on day 8^th^ of culture. After incubation, TER values were measured from all End1/E6E7 cell monolayers inserts.

Upon reaching confluence and formation of polarized monolayer (as indicated by TER values >150 Ω/cm^2^) on day 8^th^ of culture, the apical surfaces of polarized filter-grown End1/E6E7 cell monolayers were stimulated with the agonists of TLR9, CpG-ODN or RIG-I, Poly(I:C)LL (10 µg/ml) for 24 hrs. After incubation, spent media (SM) from basolateral compartment of untreated (UT-SM) or treated with CpG-ODN (CpG-SM) and Poly(I:C)LL (Poly-SM) End1/E6E7 cell monolayers were collected, spun down at 5000 g for 5 min at 4°C in a microfuge (Eppendorf) to remove any cell debris and stored in −80°C until needed for cytokine (IL-8 and IL-6) analysis by ELISA. Before putting for culture, wells containing media alone were included in all experiments as controls.

### Isolation of Peripheral Blood Mononuclear Cells

Blood samples were obtained from healthy adult blood donors aged between 21–40 years using aseptic technique into a heparinized syringe (50 U heparin/mL of blood) as described previously. Briefly, buffy coat containing peripheral blood mononuclear cells (PBMCs) were obtained after Ficoll centrifugation (750 g for 15 mins) according to the manufacturer's instructions. PBMCs were washed twice with RPMI-1640 (Invitrogen), and incubated for 1 hr at 37°C and 5% CO_2_ in RPMI supplemented with 10% FBS (Hyclone). Non-adherent cells were removed and the cells attached to plastic surface of the plate were collected as monocytes. Monocytes were incubated in ice cold DPBS containing 20 mM EDTA (Ethylenediaminetetraacetic acid, Invitrogen) for 10 minutes to detach them from surface, following which they were counted and re-plated in 24 well culture plates at a density of 5×10^4^ cells/well for further differentiation into macrophages or dendritic cells.

### Generation of monocyte derived macrophages (MDMs) and dendritic cells (MDDCs)

To obtain monocyte derived macrophages (MDMs), monocytes were cultured for 7 days in RPMI medium containing glutamax supplemented with 1% vitamins, non-essential amino acids, 1 mM sodium pyruvate, 100 µg/ml streptomycin, 100 U/ml penicillin and heat-inactivated 10% FBS, during this period monocytes would differentiate into macrophages. To obtain monocyte derived dendritic cells (MDDCs), monocytes were cultured in differentiation medium comprising RPMI 1640 supplemented with 10% FBS, 50 ng/ml of recombinant IL-4 and GM-CSF. After 5 days in differentiation medium, immature DCs were obtained and used for co-culturing with autologous T cells and End1/E6E7 cells.

### Isolation of CD3^+^ T cells

After separation of monocytes as described above, the unattached cells were used to isolate T cells. CD3**^+^** T cells were isolated with magnetic associated cell sorter (MACS) using Pan T cell Isolation Kit (Miltenyi Biotec) by employing negative selection strategy.

### Effects of End1/E6E7 cell secretions on MDMs and MDDCs

The End1/E6E7 cells were grown as polarized confluent monolayers. After formation of monolayer, cells were treated apically with either CpG-ODN or Poly(I:C)LL (10 µg/ml) for 24 hrs. Untreated cells served as controls. Spent media from basolateral compartment of UT-SM and cells stimulated with CpG-SM and Poly-SM were collected and used for incubation with MDMs or MDDCs by diluting them 1∶1 with fresh complete RPMI medium for 24 hrs.

#### (a) Stimulation of MDMs

Monocyte derived macrophages were incubated with fresh media, UT-SM, CpG-SM or Poly-SM for 24 hrs. After incubation, MDMs were challenged with LPS (100 ng/ml) for 24 hrs. Spent media was collected and stored in −80°C until cytokine analysis was performed.

#### (b) Stimulation of DC:T cell co-cultures

Immature MDDCs (2×10^4^/well) were incubated with fresh media, UT-SM, CpG-SM or Poly-SM for 24 hrs. After incubation for 24 hrs, they were stimulated with lipopolysaccharide (LPS, 100 ng/ml) along with T cells (2×10^4^/well) for 24 hrs. Supernatants were collected and stored in −80°C until analysis.

### Cytokines/chemokines analysis by Bio-Plex assay

Concentration of cytokines (IL-2, IL-4, IL-5, IL-10, IL-12 (p70), IL-13, GM-CSF, IFN-γ and TNF-α) were determined in the spent media of macrophages and DC:T co-cultures collected 24 hrs post treatment with CpG-ODN and Poly(I:C)LL by Bio-Plex™ Pro Human Cytokine Th1/Th2 assays (Bio-Rad) as per manufacturer's instructions.

### Analysis of TGF-β levels in End1/E6E7 cells and its effect on macrophages

To determine whether stimulation of End1/E6E7 cells with CpG-ODN and Poly(I:C)LL modulates the expression of TGF-β, End1/E6E7 cells were stimulated with CpG-ODN and Poly (I:C)LL (10 µg/ml) for 24 hrs. Conditioned media was collected and used to determine TGF-β levels by ELISA. In some experiments, the presence of TGF-β in the supernatants was neutralized by incubating spent media with monoclonal anti-TGF-β antibody for 1 hr at 37°C. After stimulation, cells were treated with LPS for 24 hrs and supernatants were collected for the determination of TNF-α. Similarly, to assess whether TGF-β in the conditioned medium modulates immune function of macrophages, MDMs were incubated with recombinant TGF-β (5 ng/ml) for 24 hrs. Post incubation, MDMs were treated with LPS for 2 hrs and supernatants were assayed for secretion of TNF-α.

### Statistical analysis

Statistical analysis was done using GraphPad Prism software version 5.0 (GraphPad software, CA, USA). Differences between cytokine levels were analyzed by one way analysis of variance (ANOVA) to evaluate statistical significance of differences between experimental groups with the post hoc Bonferroni test [19]. All values are expressed as the mean ± standard deviation (SD) of at least three independent experiments. The values were considered to be significant if the ‘p’ value was less than 0.05 (p<0.05).

## Results

### Ligands of TLR9 and RIG-I preparations did not contain LPS contamination

Recognizing that contamination of CpG-ODN or Poly(I:C)LL preparation with LPS is a potential problem, we tested the preparations after reconstitution in culture media to rule out LPS contamination, if any. The Limulus AmebocyteLysate (LAL) test results revealed that CpG-ODN and Poly(I:C)LL preparations contained undetectable amounts of LPS (<0.05 ng/ml) that had no effect on cytokine/chemokine secretion. We further ensured the responses observed were specific to CpG-ODN and Poly (I:C)LL by stimulating End1/E6E7 cells for 24 hrs with CpG-ODN (10 µg/mL) or Poly (I:C)LL (10 µg/mL) or combination with LPS (0.05 ng/ml). Spent media was analyzed for IL-8 levels by ELISA, which showed no change in the levels of IL-8 and the values are at par with CpG-ODN or Poly (I:C)LL treated cells (Data not shown).

### TLR9 and RIG-I ligands did not alter the viability of End1/E6E7 cells

Antiviral responses evoked by certain PRR agonists can sometime induce apoptosis as the cells seek to limit potential sites of viral replication [20]. Therefore, we sought to determine the effect of CpG-ODN and Poly(I:C)LL stimulation (10 µg/ml, 24 hrs) on the viability of End1/E6E7 cells. The results obtained by microscopic examination of trypan blue dye exclusion as well as MTT assays revealed that under the conditions tested, neither CpG-ODN nor Poly(I: C)LL affected the viability of End1/E6E7 cells as these agents did not decrease mitochondrial respiration. The viability was found to be more than 90% with both the ligands compared to the controls (Data not shown).

### End1/E6E7 cells express TLR9 and RIG-I mRNA

**Figure 1:**
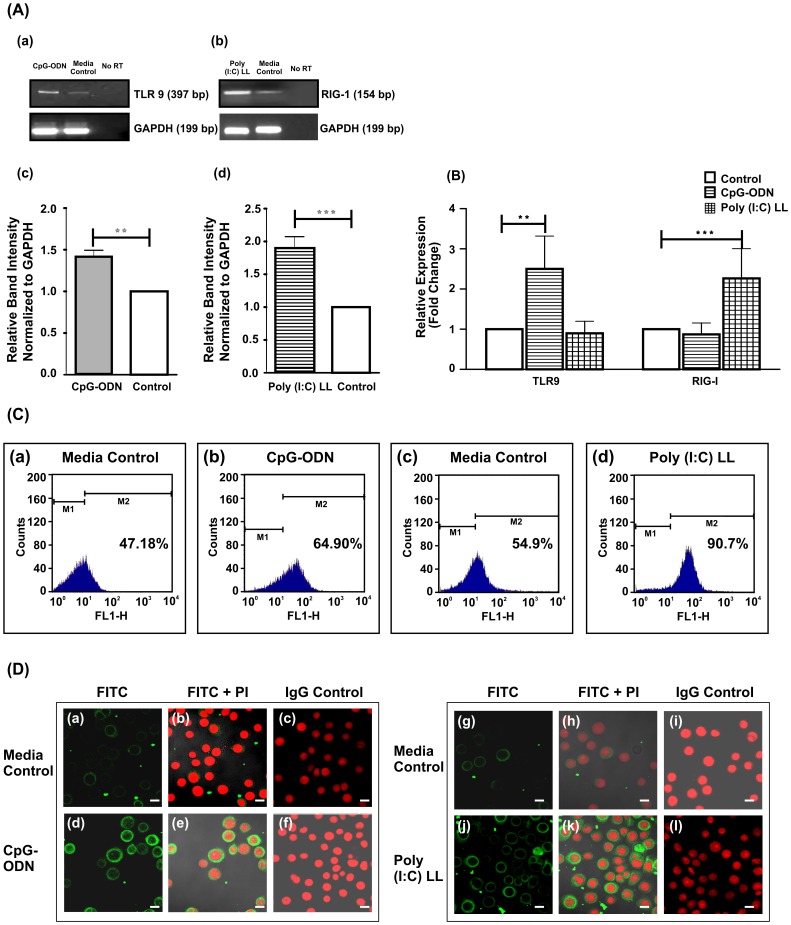
Endocervical cells (End1/E6E7) express TLR9 and RIG-I. **A. RT-PCR analysis of TLR9 and RIG-I gene expression.** (**a & b**). End1/E6E7 cells ells were seeded at a density of 1×10^5^ cells/well in a 24-well plate and treated for 6 hrs with TLR9 and RIG-I ligands (10 µg/ml). RT-PCR results show that expression of TLR9 and RIG-I mRNAs was significantly upregulated in End1/E6E7 cells upon stimulation with TLR9 and RIG-I ligands, CpG-ODN and poly (I: C)LL respectively compared to medium controls. (**c**) **&** (**d**) A quantitative densitometry analysis of TLR9 (c) and RIG-I (d) expression in End1/E6E7 cells. Values were calculated as the mean (± SD) of three separate experiments performed on different days. Level of significance (**p<0.01; ***p<0.01) was calculated by unpaired *t* test. **B. Analysis of TLR9 and RIG-I mRNA in End1/E6E7 cells by qPCR**. End1/E6E7 cells (1×10^5^) were stimulated with ligands of TLR9 (CpG-ODN: 10 µg/ml) and RIG-I {poly (I: C) LL, 10 µg/ml} for 6 hrs. Values represent mean (±SD) of three experiments performed in duplicates on different days (p<** 0.01; ***p<0.001) were calculated by ANOVA test followed by Bonferroni analysis. **C. Flow cytometry analysis of TLR9 and RIG-I expression**. Flow cytometry was used to compare the percentage of permeabilized End1/E6E7 cells labeled by anti-TLR9 and anti-RIG-I antibodies incubated with media (**a, c**) and after stimulation with their cognate ligands, CpG-ODN and Poly (I: C)LL (**b, d**) respectively. Comparison of TLR9 and RIG-I positivity was made by comparing fluorescence in the M2 channel. The figures shown are the representative pictures from three independent experiments. (FLI: log fluorescence intensity). **D. Confocal images showing the expression of TLR9 and RIG-I in End1/E6E7 cells.** Figures (a–f) End1/E6E7 cells were treated with media or CpG-ODN for 6 hrs and subsequently stained with anti-TLR9 antibody. Figures (g–l) End1/E6E7 cells were treated with media or Poly(I:C)LL for 6 hrs and subsequently stained with anti-RIG-I antibody. The figures shown are the representative picture from three independent experiments. (Magnification X 63) (Scale 10 µm).

To test whether TLR9 and RIG-I transcripts are expressed by End1/E6E7 cells, we employed a battery of assays. RT-PCR (Fig. 1 A) and qRT-PCR (Fig. 1 B) results revealed that End1/E6E7 cells constitutively expressed TLR9 and RIG-I mRNAs. Next, we looked for surface expression of TLR9 and RIG-I proteins by staining live End1/E6E7 cells and analyzing by flow cytometry. We could not detect surface expression of either of these proteins (Data not shown). However, when staining was repeated using cells that had been subjected to fixation and permeabilization as described in materials and methods, we found a significant shift in the cells fluorescence, demonstrating TLR9 and RIG-I expression could be primarily intracellular (Fig. 1 C). These results were further confirmed by immunofluorescence studies wherein we observed TLR9 and RIG-I proteins were localized in the cytoplasm of End1/E6E7 cells (Fig. 1 D a–l), which are in agreement with flow cytometry results.

### CpG-ODN and Poly(I:C)LL enhance expression TLR9 and RIG-I in End1/E6E7 cells

Having demonstrated the expression of TLR9 and RIG-I mRNA and protein in End1/E6E7 cells, we next sought to determine whether stimulation with CpG-ODN or Poly (I:C)LL modulate the expression of their cognate receptors. To accomplish this, End1/E6E7 cells were stimulated with CpG-ODN and Poly (I:C)LL (10 µg/ml) for 6 hrs. Densitometry results of RT-PCR results revealed that TLR9 and RIG-I expression was increased by 1.45 and 1.9 folds respectively after stimulation with their respective ligands as compared to controls (Fig. 1 A).The qPCR results are in good agreement with RT-PCR data (Fig. 1 B). Flow cytometry data indicated that expression of TLR9 was increased by ∼27% upon induction with CpG-ODN, whereas RIG-I expression was upregulated by ∼39% as compared to the controls (Fig. 1 C). Western blot (Fig. S1) and immunofluorescence (Fig. 1 D) studies demonstrated that expression of TLR9 and RIG-I are increased in End1/E6E7 cells after stimulated with CpG-ODN and Poly (I:C)LL.

### CpG-ODN and Poly(I:C)LL stimulation lead to increased expression of pro-inflammatory cytokines (IL-6 and IL-8)

**Figure 2:**
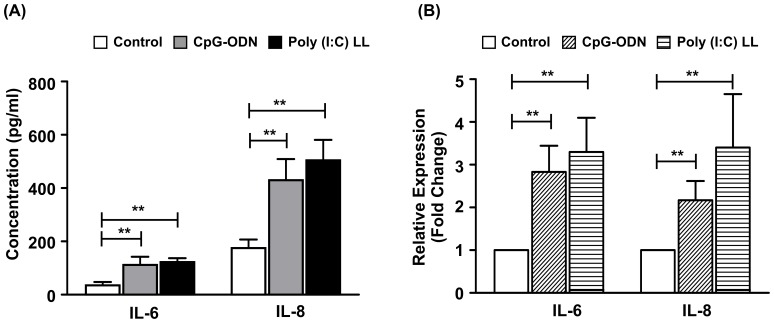
CpG-ODN and Poly (I: C)LL induce increased expression of IL-6 and IL-8. (A) End1/E6E7 cells were stimulated with CpG-ODN and poly (I: C)LL (10 µg/ml) for 6 hrs. Conditioned media was collected for quantification of IL-6 and IL-8 by ELISA. Each bar represents mean (± SD) of three separate experiments performed on different days. Level of significance (**p<0.01) was calculated by ANOVA test followed by Bonferroni analysis. (B) End1/E6E7 cells (1×10^5^ cells/well) were stimulated with ligands of TLR9 (CpG-ODN: 10 µg/ml) and RIG-I {poly (I: C) LL, 10 µg/ml} for 6 hrs. Values represent mean (±SD) of three experiments performed in duplicates on different days (**p<0.001).

Since CpG-ODN and Poly(I: C)LL are the ligands for TLR9 and RIG-I respectively, we anticipated stimulation of End1/E6E7 cells would up-regulate IL-6 and IL-8 expression. To test this hypothesis, End1/E6E7 cells were seeded at a density of 1×10^5^ cells/well in a 24-well plate and treated for 6 hrs with TLR9 and RIG-I ligands (10 µg/ml each). After the treatment, spent media and cells were collected for quantification of pro-inflammatory cytokines, IL-6 and IL-8 by ELISA (Fig. 2A) and qPCR (Fig. 2B) respectively. As expected, qPCR results demonstrated a significant elevation in IL-6 and IL-8 mRNA and protein expression in cells treated with CpG-ODN and Poly (I: C)LL in respect of controls.

### CpG-ODN and Poly(I:C)LL activate NF- κB

**Figure 3:**
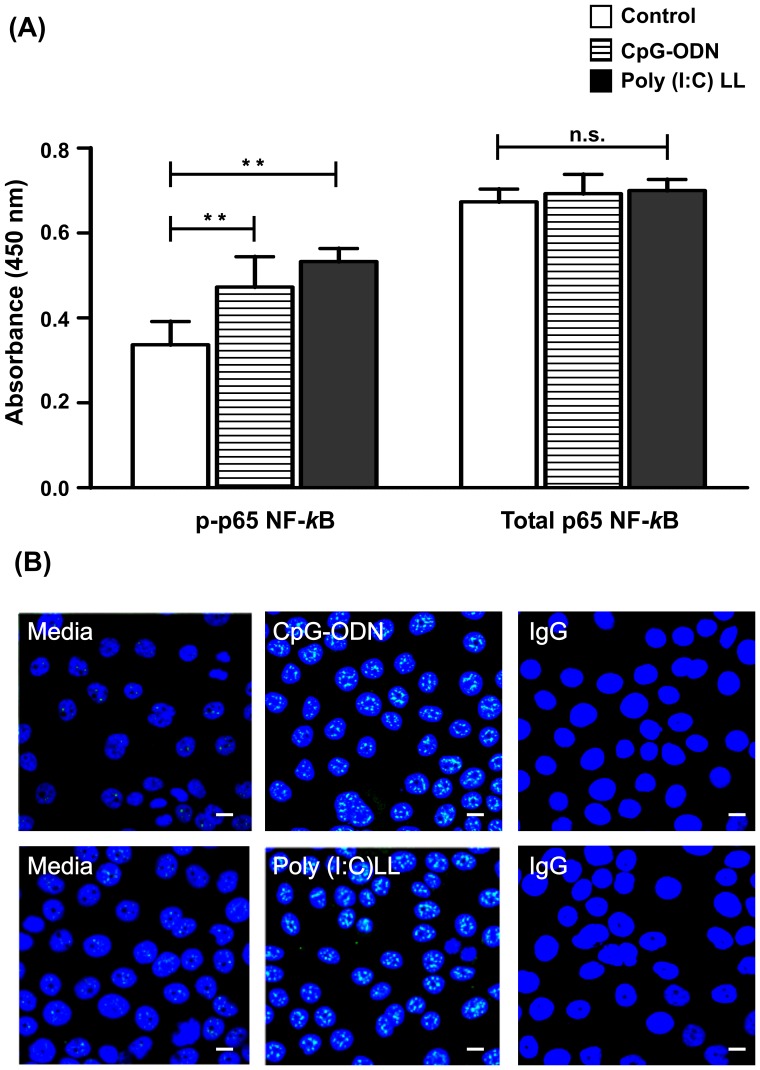
Stimulation with CpG-ODN and Poly (I: C)LL activates NF-kB pathway. (**A**). End1/E6E7 cells were seeded at a density of 1×10^5^/well in a 24-well plates and stimulated with CpG-ODN and Poly(I:C)LL (10 µg/ml for 30 min). At the end of the treatment, cells were collected, lysates were prepared and analyzed for phospho-p65-NF-kB levels. Values were calculated as the mean (± SD) of three separate experiments performed on different days. Level of significance (**p<0. 01; n.s: not significant) was calculated by ANOVA test followed by Bonferroni analysis. (**B**). Confocal images of phosphorylated p65-NF-*κ*B expression in End1/E6E7 cells before and after stimulation for 30 min with TLR9 and RIG-I ligands, CpG-ODN and Poly(I:C)LL (10 µg/ml) respectively. The images shown are the representative pictures of one of three identical experiments performed on three different days. (Magnification X 63) (Scale 10 µm).

After confirming the effect of CpG-ODN and Poly(I:C)LL on the activation of pro-inflammatory cytokines, studies were extended to examine whether these ligands had any effect in modulation of nuclear factor-κB (p65-NF-κB) expression. ELISA results indicated that stimulation of End1/E6E7 cells with CpG-ODN or Poly (I:C)LL resulted in an activation of NF-κB (Fig. 3A). Confocal data showed significant up-regulation of p65-NF-κB, and its nuclear expression indicates its role in the activation of downstream signaling required for pathogen clearance (Fig. 3B).

### Establishment of polarized End1/E6E7 cell culture

In Transwell cell culture system, epithelial cells generate an electrochemical gradient across a monolayer that reflects barrier function of the tight junctions [21], the integrity of the barrier can be measured as transepithelial resistance (TER). Epithelial cells are highly polarized as a result of the markedly different environment with which they interact at the apical (microflora) and basolateral (lamina propria) surfaces. This polarity is maintained by tight-junction complexes under normal conditions and lost under inflammatory conditions. Therefore, we chose End1/E6E7 epithelial cell line for our study as the morphological and immunocytochemical characteristics of this cell line closely resemble those of primary cells and exhibit well defined tight junctions, gap junctions similar to primary cells *in vivo* [16]. Previous studies have shown that this cell line can be used for establishment of polarized monolayer [22], [23]. Hence, we were preferentially interested in addressing ligand responsiveness in this cell line when polarized.

End1/E6E7 cells were grown to confluence on polycarbonate membrane inserts (three inserts/treatment groups, pore size 0.4 µm) till a stable TER reading was obtained. To get stable cell monolayer integrity, we monitored the culture every day for TER using a Millicell-ERS ohmmeter for a period of 8–10 days. Within this period, cells reached confluence and formed tight junctions as indicated by TER values ∼150 Ω/cm^2^/insert (background resistance 70 Ω/cm^2^/insert) (data not shown).

### CpG-ODN and Poly(I:C)LL did not affect monolayer integrity

The effect of CpG-ODN and Poly(I:C)LL ligands on monolayer integrity and barrier function was determined by measuring changes in TER values of polarized End1/E6E7 monolayers. CpG-ODN and Poly (I:C)LL (10 µg/ml) placed in the apical compartment and incubated for 24 hrs, had no effect on TER relative to medium controls, indicating maintenance of intact monolayers (Fig. S2).

**Figure 4:**
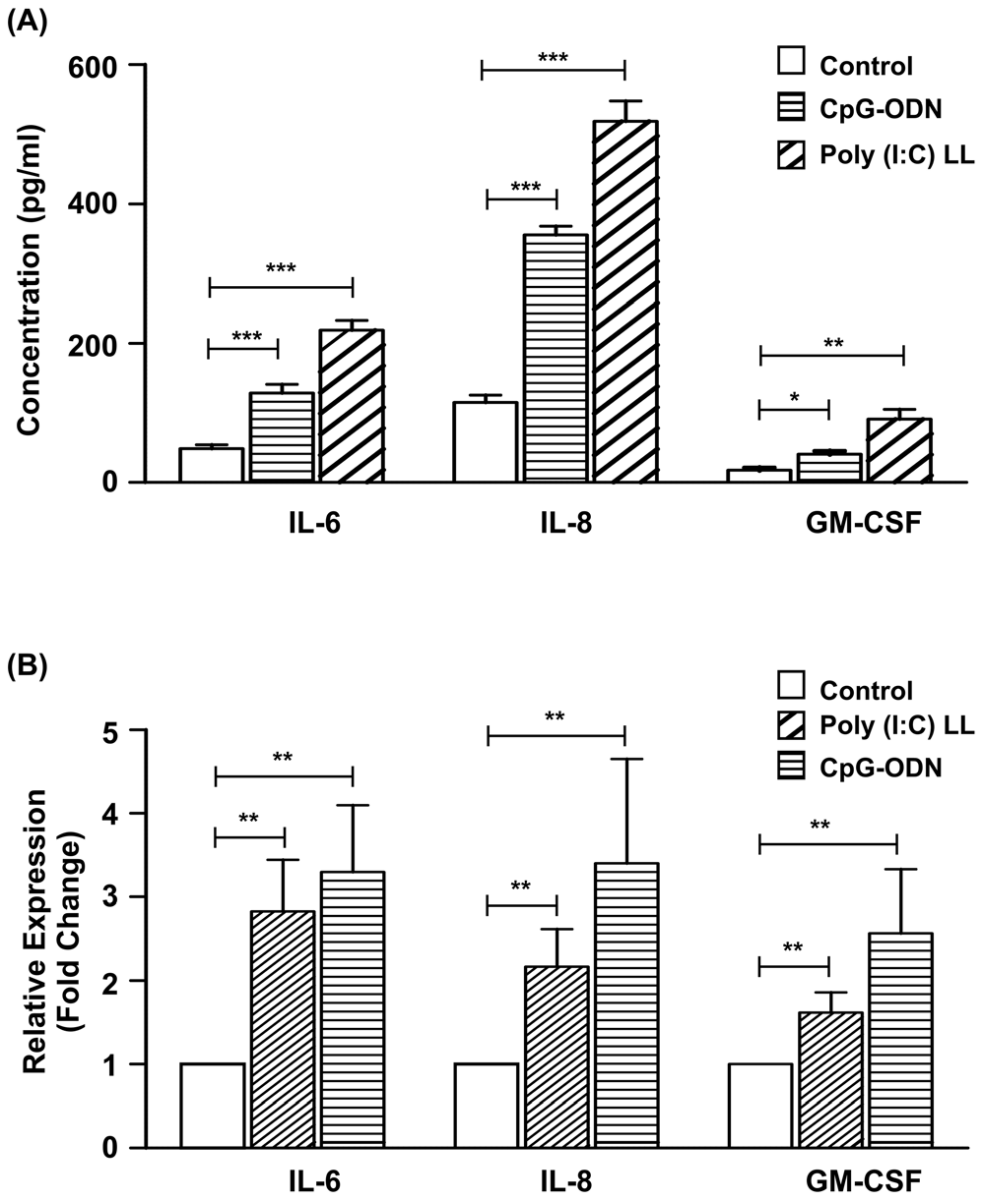
Polarized endocervical cells respond to apical stimulation with CpG-ODN and poly (I: C) LL. (A) End1/E6E7 cell monolayers were stimulated apically with CpG-ODN or Poly (I: C)LL (10 µg/ml) for 24 hrs. Following incubation, spent media from basal chamber was collected IL-6, IL-8 and GM-CSF concentrations were determined by ELISA. Each bar is the mean (± SD) of six individual observations obtained from three independent experiments. Level of significance (*p<0.5; **<0.01; ***p<0.001) was calculated by ANOVA test followed by Bonferroni analysis. (B) End1/E6E7 cell monolayers were treated apically with CpG-ODN or Poly(I:C)LL (10 µg/ml) for 4 hrs, following RNA was extracted. Abundance of transcripts of IL6, IL8 and GM-CSF was determined by qPCR. Values represent mean (±SD) of three experiments performed in duplicates on different days (p<** 0.01)were calculated by ANOVA test followed by Bonferroni analysis.

In order to determine whether stimulation of End1/E6E7 monolayers with CpG-ODN and Poly(I:C)LL initiate inflammatory responses, monolayers on day 8^th^ of culture were stimulated apically with media containing CpG-ODN or Poly(I:C) LL for 24 hrs. Following incubation, spent media from basal chamber was collected and used for quantification of IL-6, IL-8 and GM-CSF by ELISA (Fig. 4A). In some cases, cells were collected to analyze transcript levels of IL6, IL8 and GM-CSF by qPCR (Fig. 4B). There was a significant increase in the levels of IL-6, IL-8 and GM-CSF upon stimulation with both these ligands indicating apical stimulation of TLR9 and RIG-I can induce inflammatory response.

### Spent media of polarized End1/E6E7 cells modulate LPS-induced cytokines/chemokines secretion in macrophages

**Figure 5:**
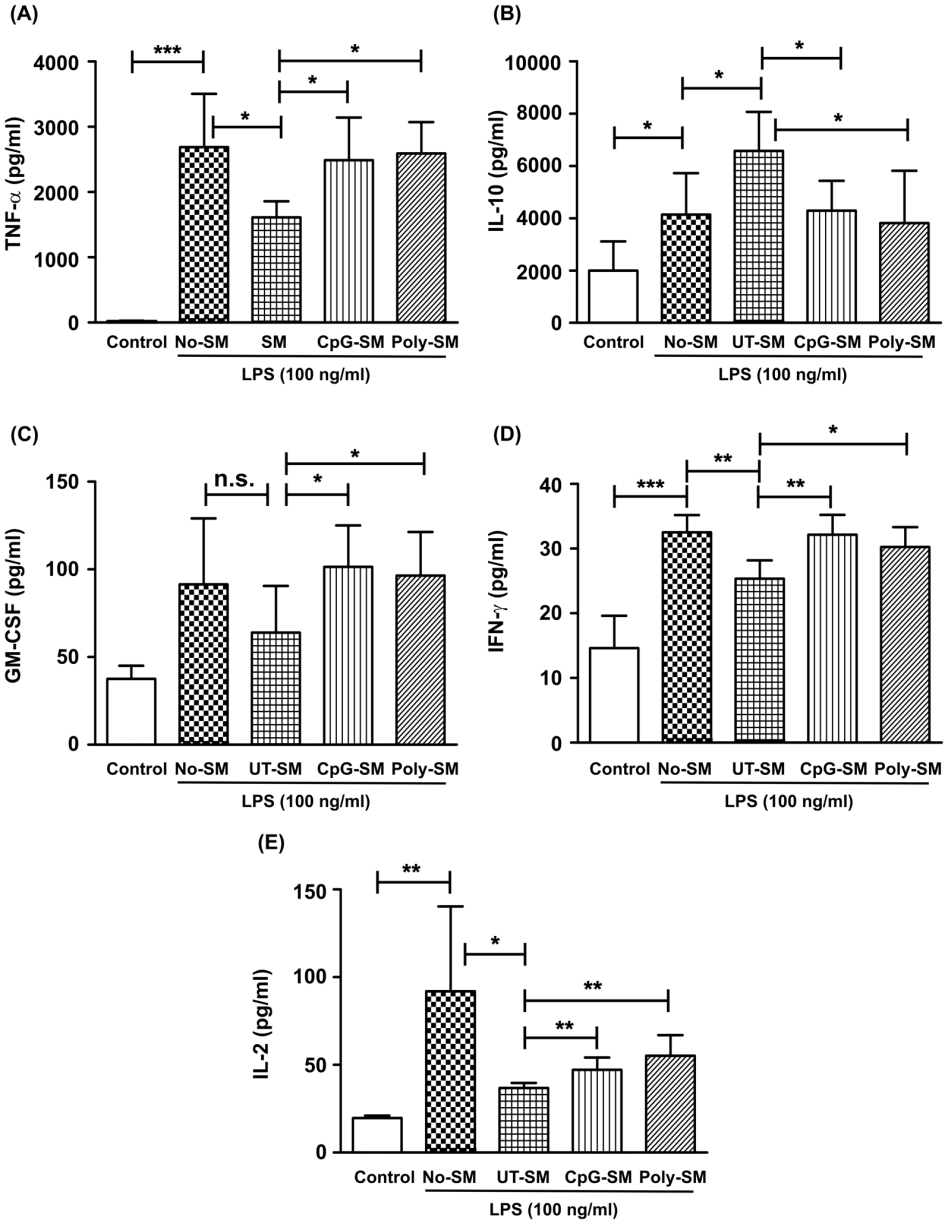
Secretions of End1/E6E7 cells modulate cytokine levels by MDMs in response to LPS. Monocyte derived macrophages (MDMs) were incubated for 24 hrs with fresh medium (No-SM) or spent media from untreated (UT-SM), CpG-ODN (CpG-SM) or poly (I: C) LL (poly-SM) treated End1/E6E7 cells. Later MDMs were stimulated with LPS (100 ng/ml) for an additional 24 hrs and supernatants were analyzed for cytokines viz., TNF-α (**A**), IL-10 (**B**), GM-CSF (**C**), IFN-γ (**D**) and IL-2 (**E**) levels Values represent mean ± SD of three experiments performed on different days. Level of significance (*p<0.05; **p<0.01; ***p<0.001; n.s: not significant) was calculated by ANOVA test followed by Bonferroni analysis.

We next examined the effect of spent medium of either unstimulated (steady state) or ligand treated End1/E6E7 cells on response of primary monocyte derived macrophages (MDMs) to LPS stimulation. For this purpose, End1/E6E7 cells were grown on culture inserts. After formation of polarized monolayers, cells were treated apically with CpG-ODN or Poly(I:C)LL for 24 hrs. Then, spent media from basal chambers of untreated (UT-SM), CpG-ODN treated (CpG-SM) and Poly(I:C)LL treated (Poly-SM) cells were collected. MDMs were incubated with spent media of End1/E6E7 cells (UT-SM, CpG-SM or Poly-SM) for 24 hrs. These cells were then stimulated with LPS for an additional 24 hrs and the supernatants were collected for cytokine/chemokine analysis. The results show that conditioned media of MDMs secreted abundant amounts of pro-inflammatory cytokines viz. TNF-α, IFN-γ, GM-CSF and IL-2. In contrast, incubation of MDMs with spent medium of untreated End1/E6E7 cells (UT-SM) significantly reduced the secretion of these pro-inflammatory cytokines and produced higher level of anti-inflammatory cytokine, IL-10 (Figure 5A–E).Interestingly, spent media of ligands stimulated End1/E6E7 cells (CpG-SM and Poly-SM) significantly increased the pro-inflammatory cytokine levels from MDMs. Thus, these results indicate that under steady state, End1/E6E7 cells restrict production of pro-inflammatory cytokines by macrophages. On the contrary, when infection is detected via TLR9 and RIG-I, the restriction is reversed and pro-inflammatory response of the macrophages is restored.

### Phagocytosis of U937 cells enhanced by spent media of End1/E6E7 cells

Phagocytosis serves as one of the key processes involved in development, maintenance of tissue homeostasis, as well as in eliminating pathogens from the host [24]. Therefore, in the current study, we asked whether End1/E6E7 cells alter phagocytic functions of U937 cells. For this purpose, fresh medium (control) and spent media obtained from End1/E6E7 cells after induction with CpG-ODN, Poly(I:C)LL ligands (UT-SM, CpG-SM or Poly-SM) was incubated with monocyte derived U937 cells for 3 hrs following which ability of these cells to phagocytose FITC labeled *E. coli* cells was determined by flow cytometry.

**Figure 6:**
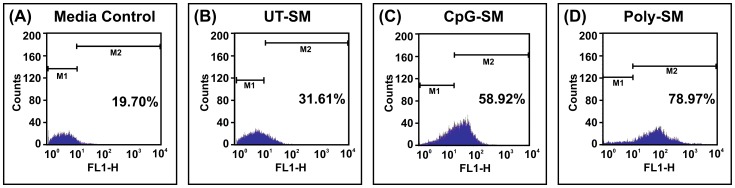
Secretions of End1/E6E7 cells enhance phagocytosis. U937 cells were incubated for 3(control) (A), untreated spent media (UT-SM) of End1/E6E7 cells (B) or spent media obtained from CpG-ODN (C) and poly(I :C)LL (D) treated End1/E6E7 cells. Quantitative analysis of U937 cells with FITC-labeled *E. coli* was performed by flow cytometry.

Quantitative analysis of U937 cells containing FITC-labeled *E. coli* was performed on a FACS vantage flow cytometry. Flow cytometry data indicated that the number of U937 with engulfed E. coli significantly increased upon incubation with spent media as compared to media control. Incubation of U937 cells with CpG-SM or Poly-SM led to further increase in phagocytosis (Fig. 6). The internalized FITC labeled *E. coli* could be observed in z stack images under confocal microscope (Fig. S3).The phagocytosis index (PCI) of medium control was ∼1.28±0.39% as compared to ∼2.05±0.51% in CpG-SM and to ∼2.60±0.55% in Poly-SM treated cells (Data not shown).

### Spent media of End1/E6E7 cells induce chemotaxis

**Figure 7:**
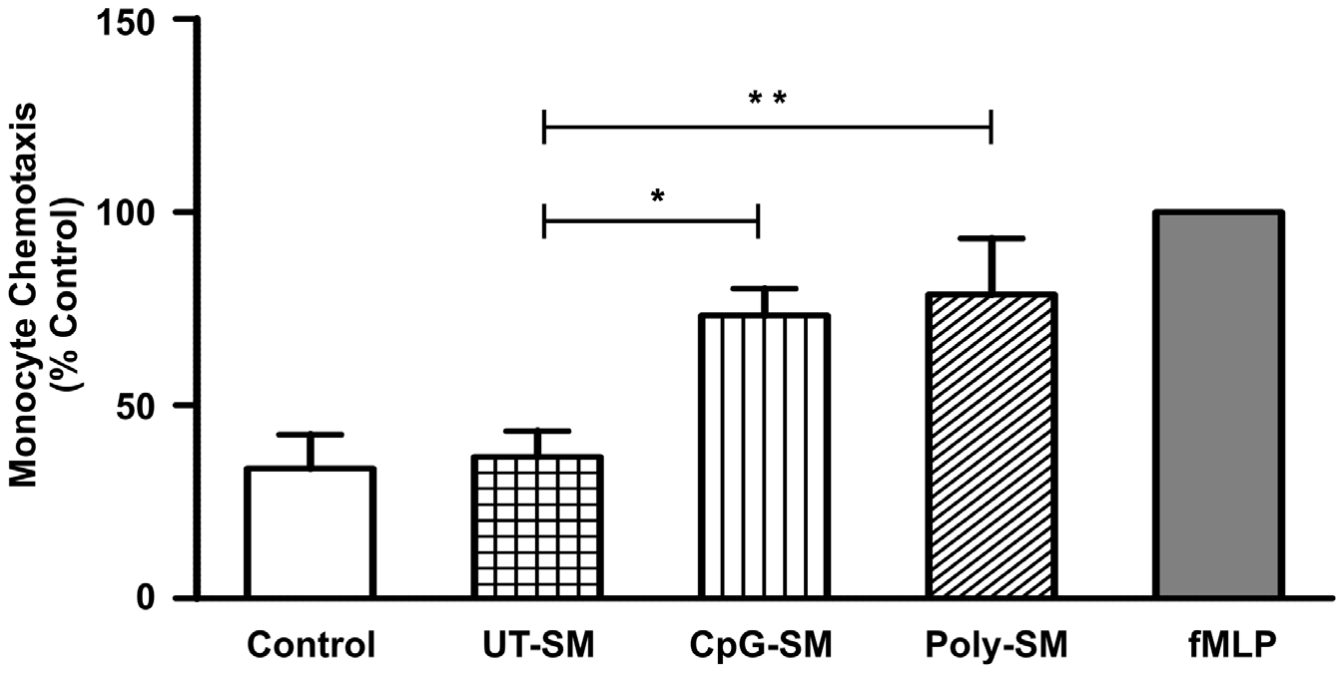
Secretions of ligand stimulated End1/E6E7 cells enhance chemotaxis of U937 cells. Spent media of End1/E6E7 cells stimulated with CpG-SM and Poly-SM were used in the lower Transwell chamber, and U937 cell were placed on the upper Transwell chamber. The data were normalized to U937 chemotaxis observed in response to 0.1 mM (N-formyl-methionyl-leucyl-phenylalanine (fMLP) in PBS-BSA, which was used as positive control (100%). Each bar is the mean ± SD from six individual observations obtained from three independent experiments. Level of significance (*p<0.05; **P<0.01) was calculated by ANOVA test followed by Bonferroni analysis.

The effect of spent media obtained from End1/E6E7 cells after stimulation with CpG-ODN (CpG-SM) and Poly(I:C)LL (Poly-SM) was analyzed *in vitro* using Boyden chamber Transwell migration assay. The results indicated that significant number of U937 cells (∼70%) migrated in response to spent media of ligand treated cells as compared to media control and UT-SM. fMLP was used as positive control (Fig. 7).

### End1/E6E7 cells modulate DC/T cell response to LPS

**Figure 8:**
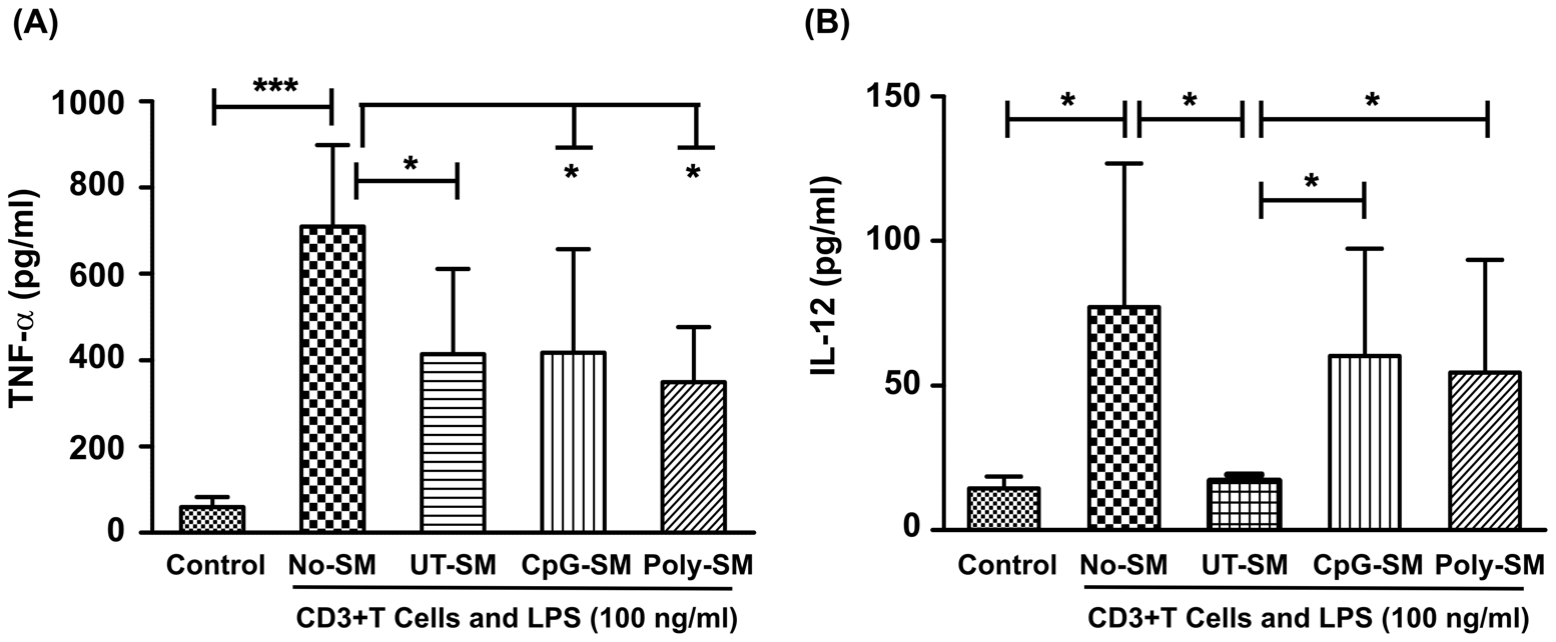
Secretions of End1/E6E7 cells modulate cytokine levels by DC: T cell-co-cultures in response to LPS. Monocyte derived dendritic cells (MDDCs) were incubated with fresh medium (No SM) or UT-SM, CpG-SM or Poly-SM for 24 hrs. Later MDDCs were stimulated with LPS (100 ng/ml) and autologous CD3**^+^** T cells for 24 hrs and spent media were analyzed for TNF-α (**A**) and IL-12 (**B**). Values represent mean ± SD of three experiments performed on different days. Level of significance (*p<0.05; ***p<0.001) was calculated by ANOVA test followed by Bonferroni analysis.

Dendritic cells are potent inducers of T cell responses, they capture pathogens and present peptide antigens to T cells thereby activate cellular immune responses. Increasing evidence suggests that cross-talk between epithelial cells and DC's would tightly regulate DC's functions in steady state. The End1/E6E7 cells are known to express multiple immmuno-modulatory factors, though detailed information on how they influence DC functions is unclear. Therefore, to unravel this, our aim was to investigate whether spent media of End1/E6E7 cells modulate response to DC/T cell co-culture. Spent media from untreated (UT-SM) and treated with CpG-ODN (CpG-SM) or Poly(I:C)LL (Poly-SM) End1/E6E7 cells were incubated with human primary MDDC's for 24 hrs. Immediately after incubation, MDDC's were incubated with LPS and CD3**^+^**T cells for an additional 24 hrs. Supernatants were collected from DC/T cell co-cultures and used for quantitation of cytokines/chemokines by Bio-Plex cytokine assay. In contrast to MDMs, spent media from unstimulated End1/E6E7 cells (UT-SM) significantly reduced secretion of TNF-α and IL-12 as compared to cultures in control group. However, spent media from ligand treated End1/E6E7 cells (CpG-SM and Poly-SM) did not restore secretion of TNF-α, although, secretion of IL-12 was significantly elevated than cultures of UT-SM group (Fig. 8 A, B).

### TGF-β regulates TNF-α from LPS-induced macrophages

**Figure 9:**
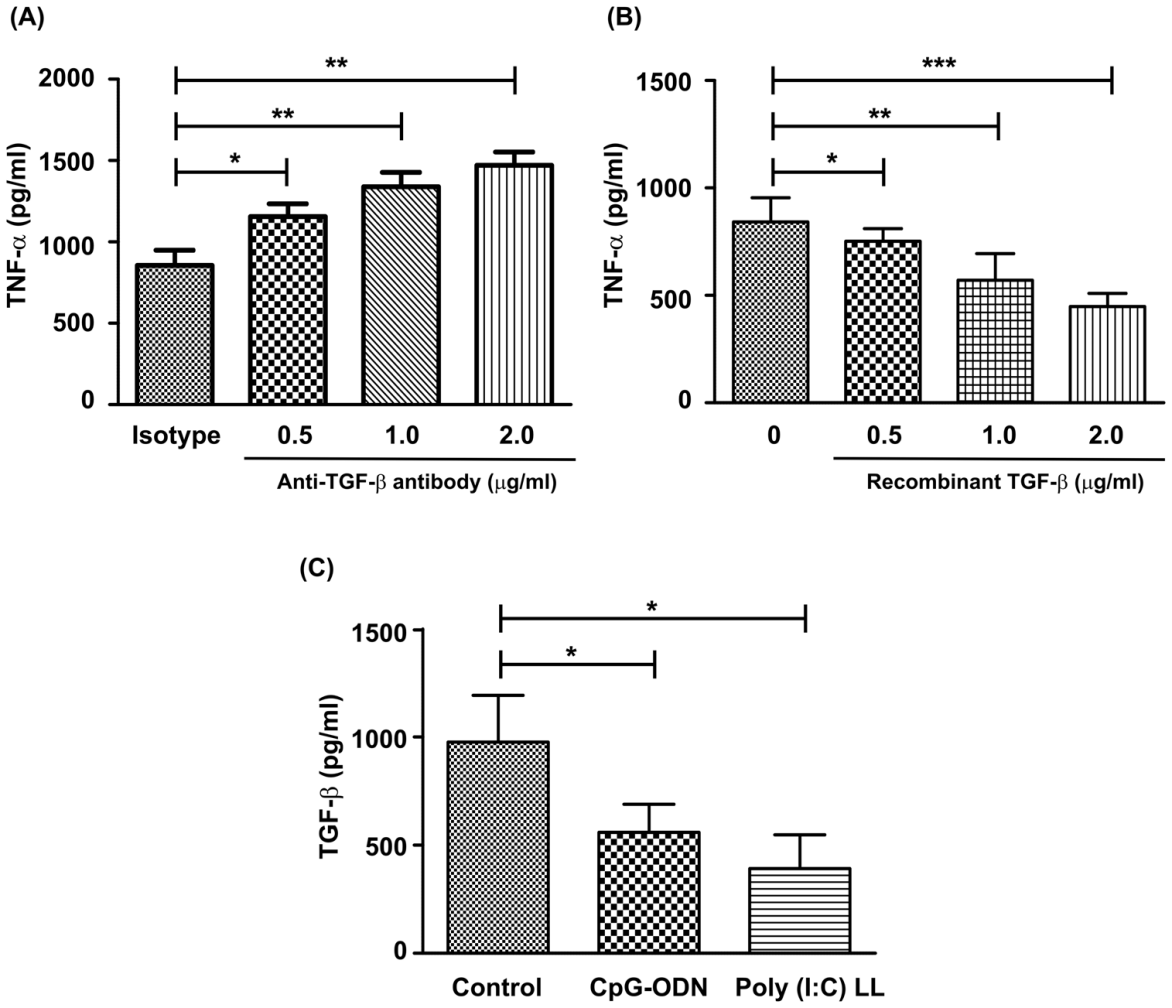
Epithelial cell derived TGF-β mediates anti-inflammatory effects on MDMs. (A) Spent media (UT-SM) was incubated with anti-TGF-β antibody at varying concentrations for 1 hr at 37°C and was then incubated with MDMs for 24 hrs. Post incubation, MDMs was stimulated with LPS for 24 hrs and secretion of TNF-α was quantitated by ELISA. (B) MDMs were stimulated with recombinant human TGF-β (0.5–2 µg/ml) for 24 hrs and further stimulated with LPS for 24 hrs. Secretion of TNF-α was quantitated by ELISA. Treatment with recombinant TGF-β decreased secretion of TNF-α from MDMs in a dose dependent manner. (C) Spent media of End1/E6E7cells stimulated with CpG-ODN or Poly-(I:C)LL were analyzed for secretion of TGF-β after 24 hrs of stimulation. Values represent mean ± SD of three experiments performed on different days Level. Of significance (*p<0.05; **p<0.01; *** p<0.001 was calculated by ANOVA test followed by Bonferroni analysis.

Our results so far revealed that at steady state, End1/E6E7 cells reduce the production of pro-inflammatory cytokines in macrophages as well as DC's. Previous reports suggest a role for epithelial derived TGF-β in down-regulating TNF-α secretion from LPS–induced macrophages [25]. To test whether TGF-β plays a similar role in our study, End1/E6E7 cells supernatants were incubated with monoclonal antibody against TGF-β and used for incubation with monocyte derived macrophages (Figure 9 a–c).After incubation, cells were treated with LPS for 24 hrs and supernatants were collected for determination of TNF-α post-stimulation. Results revealed that neutralization of TGF-β significantly increased TNF-α secretion from LPS stimulated macrophages. Similarly, addition of recombinant TGF-β reduced TNF-α production in a dose dependent manner in LPS stimulated macrophages, attributing that the levels of TGF-β in the conditioned medium modulates immune function of macrophages. These observations suggested that stimulation of End1/E6E7 cells with CpG-ODN and Poly(I:C)LL modulates expression of TGF-β. In order to test this possibility, we stimulated End1/E6E7 cells with CpG-ODN and Poly (I:C)LL for 24 hrs and the supernatants were assayed for TGF-β levels by ELISA. The results revealed that stimulation of End1/E6E7 cells with ligands significantly reduced secretion of TGF-β.

## Discussion

The female reproductive tract (FRT) is one of the primary routes of entry to the body for invading pathogens [26]. Studies have shown that the lower FRT (eg: endocervix) is periodically exposed to various infectious pathogens including viruses during sexual intercourse [27]. PRRs expressed by FRT cells play an important role in initiating innate immune responses against invading pathogens, and lack and/or poor expression of PRRs detrimentally reduce the immune responses to pathogens [18].

It is documented that viruses infecting FRT contain either DNA or express dsRNA during part of their life cycle. Therefore, intracellular recognition of non-self nucleic acids by TLR9 and RIG-I plays an important role in initial anti-viral immune response. Indeed, *in vivo* studies in mice have shown the protective role of TLR9 against genital HSV infections [28]. We have previously demonstrated that the TLR system to be a pathway for End1/E6E7 cell activation that might link to vaginal infections [18]. In the present study the expression of TLR9 and RIG-I receptors in End1/E6E7 cells was evaluated and confirmed by using a battery of assays and our results complements and extends our previous study on innate immune receptors in End1/E6E7 cells by showing expression for TLR9 and RIG-I. The RT-PCR and qPCR results demonstrated the expression of mRNA and functional receptors ofTLR9 and RIG-I by End1/E6E7 cells, and the expression of transcripts for these receptors was significantly (p<0.001) upregulated by their cognate ligands, CpG-ODN and Poly(I:C)LL respectively. Flow cytometry and immunofluorescence data also suggests that expression of both TLR9 and RIG-I was mostly intracellular and upregulated by their ligands. Additionally, stimulation of End1/E6E7 cells with the corresponding agonists, CpG-ODN and Poly(I:C)LL, induces cytokine and chemokine release. Our results are thus in agreement with those of Schaefer et al [21].

It is known that epithelial cell membrane is divided into two domains: apical and basolateral. With an apical surface to the lumen and a basolateral surface to the basement membrane and underlying epithelial cells have a structural and often functional, polarized orientation [30]. These two membrane domains serve very different functions and therefore have almost completely different compositions. These differences in the structure and composition of the apical and basolateral cell membranes might have implications for the endocytosis of the invading pathogens. This permits epithelial cells to respond to different stimuli and serve as a directional conduit for different factors. For example, in intestinal epithelial cells, TLR5, the innate immune receptor for bacterial flagellin, is preferentially expressed on the basolateral side [31]. In other studies, uterine epithelial cells were shown to secrete cytokines such as transforming growth factor-β (TGF-β) preferentially at the basolateral surface and tumor necrosis factor-α (TNF-α) at the apical surface [32].

Polarity of epithelial cells plays a crucial role in the infections by pathogens. *Neisseria gonorrhea* which infects endocervical cells enters via apical surface and is transcytosed across the epithelium and exit through the basolateral membrane without compromising the epithelial cell integrity [33].Studies investigating infection by Herpes Simplex virus (HSV-1 and 2) have shown that HSV infection in different epithelial cell lines to be domain specific. The polarized epithelial cells were 40–50 fold more susceptible to HSV infection at the apical surface than at the basolateral surface. Also, infection from the apical but not the basolateral surface led to nuclear transport of viral capsids and viral gene expression [34].

In the mucosal sites such as lung and gut, basolateral secretions of polarized epithelial cells have been shown to regulate maturation and functions of DCs, MDMs [35]. For example, airway epithelial cells inhibited secretion of TNF-α and IL-12 by monocytes, macrophages and DCs upon stimulation with LPS. Similarly, secretions from these epithelial cells also inhibited T cell proliferation via secretion of TGF-β [36], [37]. Another study from Wang et al. [37], revealed that in contrast to unstimulated epithelial cells, cells infected with respiratory synctial virus promoted T cell proliferation [36] suggesting thatsignals sent out by epithelial cells can change when an infection is sensed by them. Therefore, we reasoned that by culturing the End1/E6E7 cells to high transepithelial resistance (TER) on cell inserts, the effects of basolateral secretions (spent medium) can be studied in greater detail.

In the present study our findings suggest that End1/E6E7 cells can be grown as a polarized monolayer and the ability of these cells to respond to the ligands of TLR9 and RIG-I was retained even after polarization. We observed that at steady state, spent medium of unstimulated End1/E6E7 cells (UT-SM) reduced secretion of pro-inflammatory cytokines such as TNF-α, IFN-γ, GM-CSF and IL-2 by monocyte derived macrophages (MDMs). Conversely, MDMs secreted significantly higher amounts of anti-inflammatory cytokine IL-10. On the other hand, spent medium from End1/E6E6 cells stimulated with ligands of TLR9 and RIG-I supported a pro-inflammatory profile of cytokine secretion by macrophages. These studies collectively demonstrated the importance of epithelial and macrophage/DC interaction in the initiation of antiviral immunity. Besides, we also demonstrated that the culture supernatants of CpG-ODN or Poly(I:C)LL could induce the activation of U937 cells diffused across upper chamber to the lower chamber as shown in a Transwell migration assay.

Phagocytosis of pathogens is an essential component of host defense against microbial pathogenesis. Therefore, we tested whether spent medium from End1/E6E7 cells could affect phagocytosis. It was observed that at steady state, spent medium of End1/E6E7 cells did not affect phagocytosis of U937 cells. However, U937 cells incubated with spent medium from ligand stimulated End1/E6E7 cells, phagocytosis of U937 cells was significantly elevated. Besides, we also observed that the spent media obtained from CpG-ODN (CpG-SM) or Poly(I:C)LL (Poly-SM) stimulated End1/E6E7 cells enhanced chemotaxis of U937 cells, suggesting involvement of TLR9 and RIG-I in the recruiting immune cells to the site of infection. In line with our data, Niimi et al [29] have shown that culture supernatants of Poly (I:C) stimulated bronchial smooth muscle cells (BSMCs) increase the chemotactic activity of isolated human eosinophils.

TGF-β is a pleiotropic cytokine which plays an important role in maintaining tolerogenic environment at the gut and respiratory system. It has been shown that TGF-β exerts its effects by suppressing T-cell proliferation and induction of regulatory T cells as well as proteolytic cleavage of adaptor protein, MyD88 in monocytes/macrophages [38]. In our study, we observed that epithelial derived TGF-β inhibited secretion of TNFα from immune cells. Earlier studies have suggested that TLR stimulation of epithelial cells decreased expression of integrins which are critical for activation of TGF-β leading to reduced amounts of active TGF-β at the site of inflammation [39]. However, in our study, we observed that production of TGF-β upon stimulation with ligands of TLR9 and RIG-I in End1/E6E7 cells.

Based on our results, we propose a model that explains the regulation of innate immunity by endocervical cells. At steady state, these endocervical cells secrete TGF-β which maintains anti-inflammatory microenvironment in the cervix. As these cells sense infection via PRRs, synthesis of TGF-β is reduced. This results in decreased levels of “active” TGF-β at the site of infection which consequently allows the immune cells to mount a pro-inflammatory response to the invading pathogens. Therefore, we speculate that endocervical cells may have the ability to recruit and activate immune cell functions through the production of cytokines/chemokines and other defense molecules (eg: antimicrobial peptides, complements etc.) in response to potential pathogens. Given the nascent state of knowledge concerning this important area, it is clear that more studies are needed to provide valuable insights into immunobiology of endocervical epithelial cells.

## Supporting Information

Figure S1 **Western blot analysis of TLR9 and RIG-1 expression.** End1/E6E7 cells were seeded at a density of 1×10^5^ cells/well in a 24-well plate and treated for 6 hrs with TLR9 and RIG-1 ligands (10 µg/ml). At the end of treatment, cells were collected; protein was extracted and Western blot was performed using anti-TLR9 (A) and anti-RIG-I (B) as detailed in material and methods section. Level of significance (*p<0.05) was calculated by ANOVA test followed by Bonferroni analysis. A quantitative assessment of TLR9 (**C**) and RIG-I (**D**) expression by densitometric analysis. Values were calculated as the mean (± SD) of three separate experiments performed on different days. Level of significance (*p<0. 05) was calculated by ANOVA test followed by Bonferroni analysis.
(TIF)

Figure S2 **Effect of CpG-ODN and Poly (I: C)LL on TER.** End1/E6E7 cell monolayers were treated on day 8^th^ of the culture with CpG-ODN and Poly (I: C) LL (10 µg/ml) for 24 hrs. Monolayer integrity was determined by measuring changes in TER of polarized End1/E6E7 monolayer. CpG-ODN and poly (I: C) LL, placed in the apical compartment, had no effect on TER relative to medium control. Values were calculated as the mean (± SD) of triplicate determinations and are representative of three separate experiments performed on different days. Level of significance (n.s: not significant) was calculated by ANOVA test followed by Bonferroni analysis.
(TIF)

Figure S3 **Laser Scanning Confocal Microscopy of** ***E.coli*** **phagocytosis by U937 cells.** Monocyte derived U937 cells were utilized in phagocytosis assay as detailed in Material and Methods and cells were observed under confocal microscope. Representative z stack image of a U937 cell with engulfed FITC labeled *E. coli*. Also shown are positions of plasma membranes (◂) and labeled bacteria internalized by U937 cells (↑). (Magnification X 63) (Scale – 10 µm).
(TIF)

## References

[1] Lamont RF, Sobel JD, Akins RA, Hassan SS, Chaiworapongsa T, et al (2011) The vaginal microbiome: New information about genital tract flora using molecular based techniques. BJOG 118: 533–49. 21251190 10.1111/j.1471-0528.2010.02840.x PMC3055920

[2] Wira CR, Fahey JV, Sentman CL, Pioli PA, Shen L (2005) Innate and adaptive immunity in female genital tract: cellular responses and interactions. Immunol Rev 206: 306–35. 16048557 10.1111/j.0105-2896.2005.00287.x

[3] Andersen JM, Al-Khairy D, Ingalls RR (2006) Innate immunity at the mucosal surface: role of toll-like receptor 3 and toll-like receptor 9 in cervical epithelial cell responses to microbial pathogens. Biol Reprod 74: 824–831. 16421230 10.1095/biolreprod.105.048629

[4] Kato H, Sato S, Yoneyama M, Satoshi Y, Kosuke U, et al (2005) Cell type-specific involvement of RIG-I in antiviral response. Immunity 23: 19–28. 16039576 10.1016/j.immuni.2005.04.010

[5] Kim JG, Lee SJ, Kagnoff MF (2004) Nod1 Is an Essential Signal Transducer in Intestinal Epithelial Cells Infected with Bacteria That Avoid Recognition by Toll-Like Receptors. Infect Immun 72: 1487–1495. 14977954 10.1128/IAI.72.3.1487-1495.2004 PMC356064

[6] Kawai T, Akira S (2006) TLR signaling. Cell Death Differ 13: 816–25. 16410796 10.1038/sj.cdd.4401850

[7] Meylan E, Tschopp J (2006) Toll-like receptors and RNA helicases: two parallel ways to trigger antiviral responses. Mol Cell 22: 561–569. 16762830 10.1016/j.molcel.2006.05.012

[8] Kawai T, Akira S (2005) Pathogen recognition with Toll-like receptors. Curr Opin Immunol 17: 338–44. 15950447 10.1016/j.coi.2005.02.007

[9] Herbst-Kralovetz MM, Quayle AJ, Ficarra M, Greene S, Rose WA, et al (2008) Quantification and comparison of toll-like receptor expression and responsiveness in primary and immortalized human female lower genital tract epithelia. Am J Reprod Immunol 59: 212–224. 18201283 10.1111/j.1600-0897.2007.00566.x

[10] Lund J, Sato A, Akira S, Medzhitov R, Iwasaki A (2003) Toll-like receptor 9-mediated recognition of Herpes simplex virus-2 by plasmacytoid DCs. J Exp Med 198: 513–20. 12900525 10.1084/jem.20030162 PMC2194085

[11] Kawai T, Akira S (2010) The role of pattern-recognition receptors in innate immunity: update on Toll-like receptors. Nature Immunol 11: 373–384. 20404851 10.1038/ni.1863

[12] Weber F, Wagner V, Rasmussen SB, Hartmann R, Paludan SR (2006) Double-stranded RNA is produced by positive-strand RNA viruses and DNA viruses but not in detectable amounts by negative-strand RNA viruses. J Virol 80: 5059–64. 16641297 10.1128/JVI.80.10.5059-5064.2006 PMC1472073

[13] Sato A, Iwasaki A (2004) Induction of antiviral immunity requires Toll-like receptor signaling in both stromal and dendritic cell compartments. Proc Natl Acad Sci U S A 101: 16274–16279. 15534227 10.1073/pnas.0406268101 PMC528964

[14] Herbst-Kralovetz MM, Pyles RB (2006) Quantification of poly (I:C)-mediated protection against genital Herpes simplex virus type 2 infection. J Virol 80: 9988–9997. 17005677 10.1128/JVI.01099-06 PMC1617314

[15] Hibma MH (2012) The immune response to papilloma virus during infection persistence and regression. Open Virology J 6: 241–248. 23341859 10.2174/1874357901206010241 PMC3547310

[16] Fichorova RN, Rheinwald JG, Anderson DJ (1997) Generation of papillomavirus-immortalized cell lines from normal human ectocervical, endocervical, and vaginal epithelium that maintain expression of tissue-specific differentiation proteins. Biol Reprod 57: 847–55. 9314589 10.1095/biolreprod57.4.847

[17] Macdonald EM, Savoy A, Gillgrass A, Fernandez S, Smieja M, et al (2007) Susceptibility of human female primary genital epithelial cells to Herpes simplex virus, type-2 and the effect of TLR3 ligand and sex hormones on infection. Biol Reprod 77: 1049–1059. 17881767 10.1095/biolreprod.107.063933

[18] Patgaonkar MS, Sathe A, Selvaakumar C, Reddy KVR (2011) A Rabbit Vaginal Cell-Derived Antimicrobial Peptide, RVFHbP, Blocks Lipopolysaccharide - Mediated Inflammation in Human Vaginal Cells *In Vitro* . Cli Vacc Immunol 18: 1632–1643. 10.1128/CVI.00411-10 PMC3187028 21865417

[19] Stoline MR (1981) The status of multiple comparisons: simultaneous estimation of all pairwise comparisons in one-way ANOVA Designs. The American Statistician (American Statistical Association) 35: 134–141.

[20] Kim CH, Ahn JH, Kang SU, Hwang HS, Lee MH, et al (2010) Imiquimod induces apoptosis of human melanocytes. Arch Dermatol Res 302: 301–306. 20033192 10.1007/s00403-009-1012-0

[21] Schaefer TM, Fahey JV, Wright JA, Wira CR (2005) Innate immunity in the human female reproductive tract: antiviral response of uterine epithelial cells to the TLR3 agonist poly (I:C). J Immunol 174: 992–1002. 15634923 10.4049/jimmunol.174.2.992

[22] Zhang T, Sturgis TF, Youan BB (2011) pH-responsive nanoparticles releasing tenofovir intended for the prevention of HIV transmission. Eur J Pharm Biopharm 79: 526–36. 21736940 10.1016/j.ejpb.2011.06.007 PMC3375322

[23] Fichorova RN, Zhou F, Ratnam V, Atanassova V, Jiang S, et al (2005) Anti-human immunodeficiency virus type 1 microbicide cellulose acetate 1,2-benzenedicarboxylate in a human in vitro model of vaginal inflammation. Antimicrob Agents Chemother 49: 323–35. 15616312 10.1128/AAC.49.1.323-335.2005 PMC538889

[24] Sharma S, Yedery RD, Patgaonkar MS, Selvaakumar C, Reddy KVR (2011) Antibacterial activity of a synthetic peptide that mimics the LPS binding domain of Indian mud crab, *Scylla serrata* Anti-lipopolysaccharide Factor (SsALF) also involved in the modulation of vaginal immune functions through NF-*k*B signaling. Microb Pathog 50: 179–191. 21195157 10.1016/j.micpath.2010.12.007

[25] Smythies LE, Shen R, Bimczok D, Novak L, Clements RH, et al (2010) Inflammation anergy in human intestinal macrophages is due to Smad-induced Iκ-Bα expression and NF-κB inactivation. J Biol Chem 285: 19593–19604. 20388715 10.1074/jbc.M109.069955 PMC2885238

[26] Horne AW, Stocj SJ, King AE (2008) Innate immunity and disorders of female reproductive tract. Reproduction 135: 739–749. 18502890 10.1530/REP-07-0564

[27] Heinonen PK, Teisala K, Punnonen R, Miettinen A, Lehtinen M, et al (1985) Anatomic sites of upper genital tract infection. Obstet Gynecol 66: 384–90. 3160987

[28] Sajic D, Ashkar AA, Patrick AJ, McCluskie MJ, Davis HL, et al (2003) Parameters of CpG oligodeoxynucleotide-induced protection against intravaginal HSV-2 challenge. J Med Virol 71: 561–568. 14556270 10.1002/jmv.10518

[29] Niimi K, Asano K, Shiraishi Y, Nakajima T, Wakaki M, et al (2007) TLR3- mediated synthesis and release of eotaxin-1/CCL11 from human bronchial smooth muscle cells stimulated with double-stranded RNA. J Immunol 178: 489–495. 17182588 10.4049/jimmunol.178.1.489

[30] Puthenveedu MA, Bruns JR, Weisz OA, Linstedt AD (2003) Basolateral Cycling Mediated by a Lumenal Domain Targeting Determinant. Mol Biol Cell 14: 1801–1807. 12802056 10.1091/mbc.E02-10-0692 PMC165078

[31] Hershberg RM (2002) The epithelial cell cytoskeleton and intracellular trafficking-V. Polarize compartmentalization of antigen processing and Toll-like receptor signaling in intestinal epithelial cells. Am J Physiol Gastrointest Liver Physiol 283: 833–839. 10.1152/ajpgi.00208.2002 12223342

[32] Grant KS, Wira CR (2003) Effect of mouse uterine stromal cells on epithelial cell transepithelial resistance (TER) and TNF-a and TGF-b release in culture. Biol Reprod 69: 1091–1098. 12773432 10.1095/biolreprod.103.015495

[33] Hopper S, Wilbur JS, Vasquez BL, Larson J, Mehr IJ, et al (2000) Isolation of *Neisseria gonorrhoeae* mutants that show enhanced trafficking across polarized T84 epithelial monolayers. Infect Immun 68: 896–905. 10639460 10.1128/iai.68.2.896-905.2000 PMC97219

[34] Galen B, Cheshenko N, Tuyama A, Ramratnam B, Herold BC (2006) Access to nectin favors Herpes simplex virus infection at the apical surface of polarized human epithelial cells. J Virol 80: 12209–12218. 17005657 10.1128/JVI.01503-06 PMC1676285

[35] Shaykhiev R, Bals R (2007) Interactions between epithelial cells and leukocytes in immunity and tissue homeostasis. J Leukoc Biol 82: 1–15. 17452476 10.1189/jlb.0207096

[36] Mayer AK, Bartz H, Fey F, Schmidt LM, Dalpke AH (2008) Airway epithelial cells modify immune responses by inducing an anti-inflammatory microenvironment. Eur J Immunol 38: 1689–1899. 18421791 10.1002/eji.200737936

[37] Wang H, Su Z, Schwarze J (2009) Healthy but not RSV-infected lung epithelial cells profoundly inhibit T cell activation. Thorax 64: 283–290. 18710906 10.1136/thx.2007.094870 PMC2655656

[38] Naiki Y, Michelsen KS, Zhang W, Chen S, Doherty TM, et al (2005) Transforming growth factor beta differentially inhibits MyD88 dependant but not TRAM and TRIF dependant lipopolysaccharide induced TLR4 signaling. J Biol Chem 280: 5491–5495. 15623538 10.1074/jbc.C400503200

[39] Takabayshi K, Corr M, Hayashi T, Redecke V, Beck L, et al (2006) Induction of a homeostatic circuit in lung tissue by microbial compounds. Immunity 24: 475–87. 16618605 10.1016/j.immuni.2006.02.008

