# Supplementary figures and images for: TLR9 and RIG-I Signaling in Human Endocervical Epithelial Cells Modulates Inflammatory Responses of Macrophages and Dendritic Cells In Vitro
Source: PLoS One. 2014 Jan 7;9(1):e83882. doi: 10.1371/journal.pone.0083882 (PMC3883652; doi:10.1371/journal.pone.0083882)

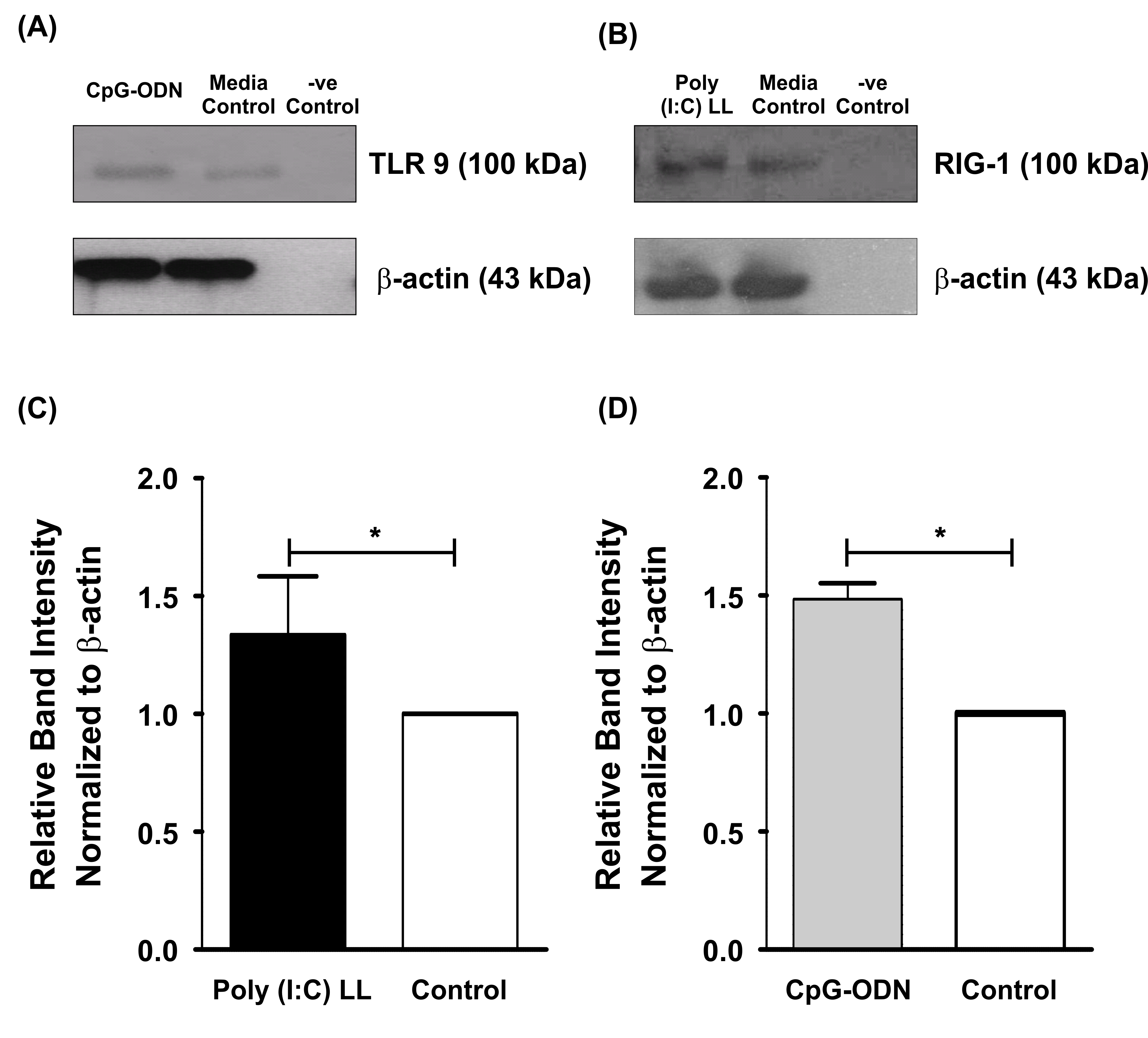

Supplement: Figure S1 — Western blot analysis of TLR9 and RIG-1 expression. End1/E6E7 cells were seeded at a density of 1×10^5^ cells/well in a 24-well plate and treated for 6 hrs with TLR9 and RIG-1 ligands (10 µg/ml). At the end of treatment, cells were collected; protein was extracted and Western blot was performed using anti-TLR9 (A) and anti-RIG-I (B) as detailed in material and methods section. Level of significance (*p<0.05) was calculated by ANOVA test followed by Bonferroni analysis. A quantitative assessment of TLR9 (C) and RIG-I (D) expression by densitometric analysis. Values were calculated as the mean (± SD) of three separate experiments performed on different days. Level of significance (*p<0. 05) was calculated by ANOVA test followed by Bonferroni analysis. (TIF) [file pone.0083882.s001.tif]

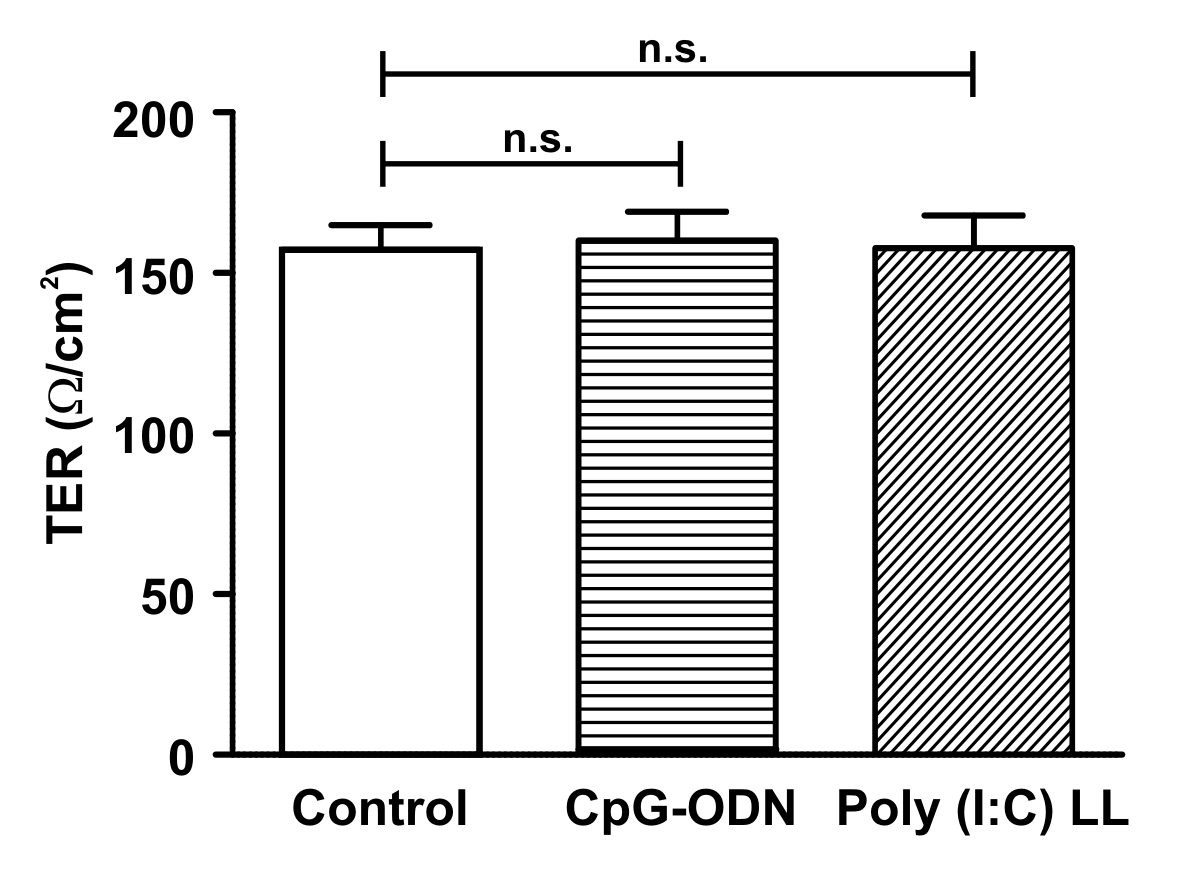

Supplement: Figure S2 — Effect of CpG-ODN and Poly (I: C)LL on TER. End1/E6E7 cell monolayers were treated on day 8th of the culture with CpG-ODN and Poly (I: C) LL (10 µg/ml) for 24 hrs. Monolayer integrity was determined by measuring changes in TER of polarized End1/E6E7 monolayer. CpG-ODN and poly (I: C) LL, placed in the apical compartment, had no effect on TER relative to medium control. Values were calculated as the mean (± SD) of triplicate determinations and are representative of three separate experiments performed on different days. Level of significance (n.s: not significant) was calculated by ANOVA test followed by Bonferroni analysis. (TIF) [file pone.0083882.s002.tif]

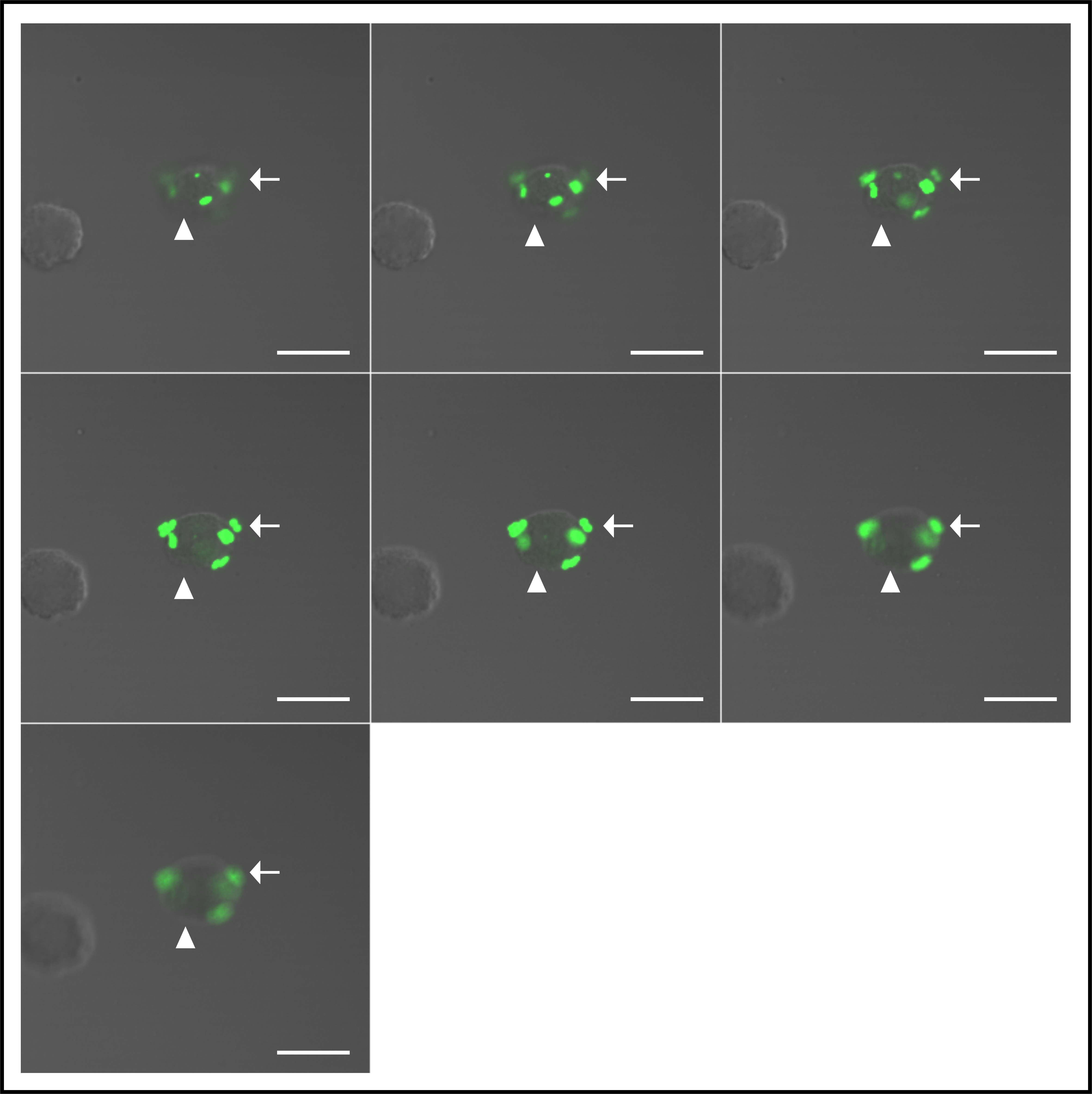

Supplement: Figure S3 — Laser Scanning Confocal Microscopy of E.coli phagocytosis by U937 cells. Monocyte derived U937 cells were utilized in phagocytosis assay as detailed in Material and Methods and cells were observed under confocal microscope. Representative z stack image of a U937 cell with engulfed FITC labeled E. coli. Also shown are positions of plasma membranes (◂) and labeled bacteria internalized by U937 cells (↑). (Magnification X 63) (Scale – 10 µm). (TIF) [file pone.0083882.s003.tif]
